# Acetylation of α-tubulin restores endothelial cell injury and blood–brain barrier disruption after intracerebral hemorrhage in mice

**DOI:** 10.1038/s12276-025-01454-9

**Published:** 2025-05-07

**Authors:** Xuejiao Lei, Eryi Sun, Xufang Ru, Yulian Quan, Xuezhu Chen, Qian Zhang, Yougling Lu, Qianying Huang, Yujie Chen, Wenyan Li, Hua Feng, Yang Yang, Rong Hu

**Affiliations:** 1 Department of Neurosurgery, Southwest Hospital, Third Military Medical University (Army Medical University), Chongqing, China; 2 Department of Neurosurgery, The Affiliated People’s Hospital of Jiangsu University, Zhenjiang, China; 3 Department of Pathology, Public Health Medical Center, Chongqing, China; 4 Clinical Medical Research Center, Southwest Hospital, Third Military Medical University (Army Medical University), Chongqing, China; 5 State Key Laboratory of Trauma and Chemical Poisoning, Chongqing, China; 6 Department of Neurosurgery, The 904th Hospital of PLA, Anhui Medical University, Wuxi, China

**Keywords:** Translational research, Stroke

## Abstract

Damage to endothelial cells (ECs) is a key factor in blood–brain barrier (BBB) disruption after intracerebral hemorrhage (ICH). While microtubules are essential for EC structure, their role in BBB injury remains unclear. Here we investigated the role of acetylated α-tubulin (α-Ac-Tub) in BBB integration after ICH. Using an autologous blood injection model in the striatum, we showed that the expression of α-Ac-Tub and MEC17, an α-tubulin acetyltransferase, significantly decreased along the vessels around the hematoma after ICH. Conditional MEC17 knockout in ECs further reduced α-Ac-Tub levels and exacerbated BBB leakage, brain edema, hematoma expansion, inflammation and motor dysfunction. Conversely, selective α-Ac-Tub upregulation in ECs via intravenous delivery of AAV-BI30-MEC17-GFP alleviated BBB dysfunction and improved motor recovery. Similarly, the HDAC6 inhibitor tubastatin A enhanced α-Ac-Tub levels, mitigating BBB damage and neurological deficits. Mechanistically, α-Ac-Tub deficiency in ECs reduced tight junction proteins (ZO-1 and Claudin5) and increased F-actin stress fibers through RhoA activation. Together, our findings highlighted α-Ac-Tub as a therapeutic target for restoring BBB function and reducing brain injury after ICH.

## Acetylated α-tubulin restores blood–brain barrier after hemorrhage

Intracerebral hemorrhage (ICH) is a severe type of stroke with high mortality and disability rates. The blood–brain barrier (BBB), which protects the brain, often gets damaged during ICH, leading to further brain injury. This study explores how stabilizing microtubules can protect the BBB after ICH. The researchers used mice to study the effects of acetylated α-tubulin (α-Ac-Tub) on the BBB. They found that reducing α-Ac-Tub worsens BBB damage, whereas increasing it helps to protect the BBB. They used genetic techniques and a drug called tubastatin A to increase α-Ac-Tub levels in mice. This approach reduced brain damage and improved recovery after ICH. The study shows that α-Ac-Tub helps to maintain BBB integrity by preventing harmful changes in cell structure. These findings suggest that targeting α-Ac-Tub could be a new way to treat ICH and protect the brain.

This summary was initially drafted using artificial intelligence, then revised and fact-checked by the author.

## Introduction

Intracerebral hemorrhage (ICH) represents one of the most severe forms of stroke, with an estimated annual mortality of approximately 2.8 million individuals. The acute-phase mortality rate can reach up to 40%, and more than 90% of survivors endure varying degrees of neurological impairment^1,2^. After ICH, the rupture of the blood–brain barrier (BBB) surrounding the hematoma engages in brain edema and neuroinflammation, both of which are critical contributors to hematoma expansion and further neurological damage^3^. Therefore, early preservation of the BBB is crucial for mitigating secondary damage after ICH.

The BBB is composed of microvascular endothelial cells (ECs) interconnected by tight junction proteins, along with pericytes, astrocytic endfeet and the basement membrane^4^. As the primary cellular component of the BBB, ECs not only compartmentalize blood but also interact with the brain parenchyma via surface receptors and secreted cytokines. After a stroke, damage to ECs within the BBB disrupts the structural and functional integrity of the neurovascular unit, thereby contributing to unfavorable patient outcomes^5^. Previous research has demonstrated that the reorganization of the endothelial cytoskeleton plays a critical role in BBB disruption after a stroke. The rapid formation of stress fibers induces endothelial contraction and the degradation of tight junction proteins, ultimately widening intercellular gaps between ECs and increasing BBB permeability^6,7^. In addition to actin filaments, ECs receive and integrate various mechanical stimuli through cell surface receptors and utilize microtubules—another critical cytoskeletal component with mechanosensing properties—to transmit signals^8^. Microtubule-associated proteins help to regulate microtubule stability and protect cellular structures from stress^9,10^. However, the role of microtubules in ICH-induced EC injury remains largely unexplored.

Microtubules undergo a range of posttranslational modifications, including acetylation and tyrosination, with each modification playing a distinct role in regulating microtubule functionality^11^. Among these, acetylation is the sole modification that occurs within vascular microtubules and serves as an indicator of microtubule stabilization^12^. Mechanistically, acetylated α-tubulin (α-Ac-Tub) is regulated by MEC17 and HDAC6/SIRT2, maintaining the dynamic equilibrium of microtubule assembly/disassembly in normal cells^13,14^. Recent studies have demonstrated that microtubule assembly enhances EC resistance to mechanical stress, highlighting its role in preserving the structural integrity and function of ECs in ICH^15,16^. In addition, microtubule assembly is reported to inhibit stress fiber formation, potentially mitigating the degradation of tight junction proteins and thereby reducing BBB damage^17^.

The current study focused on elucidating specific roles and underlying mechanisms of α-Ac-Tub in ECs in the context of BBB impairment after ICH. Our results revealed that reduction of α-Ac-Tub in ECs exacerbates BBB permeability by disrupting the expression of tight junction proteins and facilitating the formation of stress fibers through activating RhoA after ICH. By contrast, the preservation of α-Ac-Tub levels in ECs through conditional overexpression of MEC17 in ECs and treatment with tubastatin A (TBA), a HDAC6 inhibitor, mitigates BBB damage and functional impairment after ICH by inhibiting stress fiber formation. Collectively, our findings suggest α-Ac-Tub as a potential target for restoring BBB function after ICH.

## Materials and methods

### Animals

Adult mice C57 weighing approximately 20–25 g and 8–10 weeks old were used in this study. Cdh5 Cre^ERT^ mice and MEC17^flox/flox^ mice were purchased from Cyagen Biosciences and mated to produce Cdh5 Cre^ERT^:MEC17^fl/fl^ mice. Tamoxifen (75 mg/kg body weight; 10540-29-1, Sigma) was dissolved in corn oil and intragastrically administered once every 24 h for five consecutive days to induce gene recombination in 4-week-old Cdh5 Cre^ERT^:MEC17^fl/fl^ mice. Randomization was performed by randomly grouping the mice. The mice were maintained in a humidity-controlled room (with a temperature maintained at 25 ± 1°C, and a 12-h light/dark cycle) with food and water ad libitum. All the performed experiments were compliant with the Animal Research: Reporting of In Vivo Experiments (ARRIVE) guidelines. The protocols used in this study were subjected to approval from the Laboratory Animal Welfare and Ethics Committee of Third Military Medical University (AWUWEC20210675). The experiments were performed in accordance with the guidelines of the Guide for the Care and Use of Laboratory Animals. The experimental design is shown in Supplementary Fig. 1.

### ICH model and treatment

To establish the ICH model, the mice were anesthetized using a 2% mixture of isoflurane and air (1–2 l/min). Under stereotactic guidance (RWD Life Science), a small cranial burr hole was made above the striatum (Bregma coordinates: anteroposterior +0.8 mm, mediolateral 2 mm). The autologous arterial blood was obtained by puncturing the central tail artery with a sterile needle (26 gauge) and collecting blood into an unheparinized capillary tube. The blood sample (25 μl) was transferred quickly into the glass barrel of a sterile syringe (33 gauge; Hamilton). The blood was injected into the right striatum (Bregma coordinate: dorsoventral −3 mm) at a rate of 2 μl/min with a microinfusion pump (Harvard Apparatus)^18,19^. The following animals were excluded from the analysis: (1) anesthesia-related mortality (6 mice were excluded due to deaths associated with the anesthesia process) and (2) post-ICH mortality (22 mice were excluded due to deaths after ICH). TBA (SML0044, Sigma) was first dissolved in dimethyl sulfoxide to form a 10 mg/ml stock solution, which was further diluted with saline. The mice were then administered TBA (25 mg/kg, intraperitoneally) or a corresponding equivalent of the vehicle 1% dimethyl sulfoxide) immediately after ICH^20,21^. In addition, to overexpress MEC17 (NM_001142744), the AAV-BI30-MEC17-GFP (100 μl, titer 1 × 10^13^ copies/ml; BrainVTA) was intravenously injected via the tail vein 3 weeks before the induction of ICH. The control group received a corresponding equivalent of the AAV-BI30-GFP. The brain tissue around the hematoma was collected at different time points after ICH for morphological and biochemical experiments in a double-blind manner.

### Cell culture, lentivirus transduction and drug treatment

Primary human brain microvessel ECs (HBMECs) were purchased from Cell Systems (ACBRI 376). HBMECs were grown in Clonetics EGM-2 MV medium (CC-3202, Lonza), and only up to eight passages were used for experiments. Recombinant lentivirus (rLV) vectors (rLV-Ef1a-Puro, rLV-Ef1a-MEC17-Puro and rLV-Ef1a-shRNA(MEC17)-Puro) (BrainVTA) were stably transduced into HBMECs for upregulating or downregulating the expression of MEC17. Transfected HBMECs were selected with puromycin (2 μg/ml solution) for 2 days, after which they were used for subsequent experiments. To mimic the ICH model, hemin (50 μM, HY-19424, MedChemExpress) was added into the culture medium for 24 h (ref. ^22^). The efficiency of interfering and overexpression of rLV was determined through evaluating MEC17 expression using immunoblotting. In addition, rhosin (40 μM, HY-12646, MedChemExpress) was added into the culture medium to inhibit the RhoA-GTPase, for 24 h.

### Immunofluorescence staining

The mice in each group were deeply anesthetized and perfused transcardially with 0.01 M phosphate-buffered saline (PBS) followed by 4% paraformaldehyde in 0.01 M PBS. The whole brain was isolated, post-fixed in 4% paraformaldehyde for 24–48 h and then stored in 30% sucrose in 0.01 M PBS solution for 48 h for cryoprotection. After embedding and freezing with optimal cutting temperature compound, brains were sectioned into 30-µm-thick slices using a cryostat (Leica). Brain slices or HBMEC slides post-fixed were incubated with a 3% bovine serum albumin blocking solution for 1 h, then incubated with primary antibodies overnight at 4 °C, followed by incubation with Alexa Fluor secondary antibodies for 1 h at room temperature. Nuclei were stained with DAPI (Sigma-Aldrich) for 10 min, and images were captured with a confocal microscope (Carl Zeiss, Jena) and analyzed using ZEN 2011 software (Carl Zeiss) with an installed anti-fade agent (Santa Cruz Biotechnology, sc-24941). First antibodies for immunofluorescence included rabbit anti-α-Ac-Tub (Abcam, ab179484, 1:1000); rabbit anti-MEC17 (Abcam, ab184778, 1:200); mouse anti-CD31 (Abcam, ab9498, 1:500); rabbit anti-CD31 (Abcam, ab28364, 1:500); rabbit anti-ZO-1 (Invitrogen, 61-7300, 1:50); anti-Claudin5 (Abcam, ab131259, 1:100); rabbit anti-laminin (Sigma, L9393, 1:100); mouse anti-F-actin (Abcam, ab205, 1:100); rat anti-NG2 (Invitrogen, MA5-24247, 1:100); chicken anti-GFAP (Invitrogen, PA1-10004, 1:1000); rabbit anti-IBA1 (Abcam, ab178846, 1:1000).

### Immunoblotting

Total protein from HBMECs treated with hemin and with overexpression or knockdown of MEC17, as well as brain tissues around hematoma in each group, were lysed in cold RIPA buffer (Sigma-Aldrich) and protease inhibitor cocktail (Roche). Protein concentrations were measured with the enhanced BCA Protein Assay Kit (Beyotime). Subsequently, 20 μg of protein from each sample was separated on a 10% SDS–PAGE gel and then transferred to polyvinylidene fluoride membranes (Roche). The membranes were first incubated at room temperature in TBST with bovine serum albumin and 0.05% Tween 20 for 2 h, followed by an overnight incubation at 4 °C with primary antibodies as follows: rabbit anti-MEC17 (Abcam, ab184778, 1:1,000); rabbit anti-MEC17 (Proteintech Group, 28828-1-AP, 1:1,000); rabbit anti-α-Ac-Tub (Abcam, ab179484, 1:1,000); rabbit anti-ZO-1 (Invitrogen, 61-7300, 1:1,000); anti-Claudin5 (Abcam, ab131259, 1:1,000); mouse anti-α-tubulin (CST, 3873, 1:2,000); mouse anti-F-actin (Abcam, ab205, 1:1,000); rabbit anti-β-actin (CST, 4970, 1:2,000); rabbit anti-RhoA (CST, 8789, 1:1,000), and subsequently with HRP-conjugated secondary antibodies at room temperature for 1 h. Protein bands were detected using the ChemiDoc XRS^+^ Imaging System (Bio-Rad) and the Western Bright ECL kit (Advansta). Densitometry for each membrane was performed using Image Lab software (Bio-Rad).

### In vitro BBB model and BBB permeability assay

The in vitro BBB model was established in cell culture inserts as described previously^7^. The transwell polyethylene terephthalate membranes (0.4-mm pore, 11-mm diameter; Corning) were coated with collagen (15 mg/ml) and fibronectin (30 mg/ml). HBMECs were seeded onto the membrane at a density of 2.5 × 10^5^ cells per membrane. Cultures were maintained at 37 °C in humidified 95% air and 5% CO_2_ for 4 days to reach confluence. To assess paracellular permeability after hemin treatment, Alexa 555 cadaverine (0.95 kDa), tetramethylrhodamine isothiocyanate (TRITC)-dextran (4.4 kDa; Sigma-Aldrich) or fluorescein Isothiocyanate (FITC)-dextran (70 kDa; Sigma-Aldrich) were added into the luminal chamber at a concentration of 1 μM/ml in 500 μl medium. Fluorescence intensity was measured with a fluorescence reader at 1, 2, 3, 4 and 6 h by removing 50 μl medium from the lower (abluminal) chamber. The concentrations of tracers in samples were calculated from a standard curve fit using known concentrations of tracers. Fifty microliters of fresh medium was added after each reading. Paracellular permeability was calculated by measuring the diffusion rate of tracers from the luminal to the abluminal chamber. The diffusion rate of tracers was expressed as pmol/mm^2^/min.

### Trans-endothelial electrical resistance

The barrier properties of ECs were assessed by measuring trans-endothelial electrical resistance (TEER) across confluent monolayers using an electrical cell–substrate impedance sensing device (Applied Biophysics), as previously outlined^23^. In brief, brain ECs were cultured on small gold microelectrodes (eight-well chamber slides, ECIS, Applied Biosystem) in endothelial growth medium supplemented with 5% FBS. Before experimentation, the growth medium was replaced with serum-free medium, and then a 4,000-Hz a.c. signal with 1-V amplitude was applied across the cell monolayers. Once the electrical resistance stabilized at approximately 1,000 Ω, the endothelial monolayers were monitored for 24 h. The total electrical resistance was dynamically measured across the monolayers, encompassing the resistance between the basal surface of the cells and the electrode, which reflects focal adhesion, as well as the resistance between the cells.

### Behavioral tests

#### Accelerated rotarod test

The accelerated rotarod test, as previously described by Yang et al.^24^, was conducted to assess grip strength in mice. The speed of the rotarod gradually increased from 5 to 45 rpm over 2.5 min. The time taken for the mice to fall (or cling onto and complete three full rotations with the rod) was recorded for statistical analysis. Each mouse underwent three trials, with a 10-min interval between trials.

#### Irregular ladder walking

In this test, mice from various groups were allowed to traverse a horizontal ladder measuring 100 cm in length, 19 cm in height and 10 cm in width, with irregular spacing between the rungs, following previously described procedures^21^. To prevent mice from memorizing the pattern, the irregular spacing between rungs was altered in different trials. Videos of the trials were recorded and analyzed by blinded observers. Results were expressed as the percentage of contralateral limb slips divided by the total steps. Baseline measurements were obtained by allowing the mice to walk on the ladder. Mice with more than 10 erroneous steps per 50 steps were excluded from the analysis.

#### Beam walking

Similar to irregular ladder walking test, beam walking is also used to assess the capacity of precise paw placement. Beam walking was performed on a narrow beam (0.6 cm wide, 120 cm long and 60 cm high). During training and assessments, the mice were recorded by a video camera, and paws slipping down the horizontal surface of the beam were identified as foot faults. The number of contralateral forelimb and hindlimb foot faults within 50 steps were counted and analyzed after ICH.

The following two exclusion criteria were used to exclude mice from the analysis of behavioral data. In the evaluation before ICH surgery, mice that slipped more than 10 times per 50 steps in beam walking and ladder rung walking were excluded because they were unable to learn the behavioral tasks. In the evaluation after ICH, mice that walked fewer than 50 steps were excluded because they failed to complete these behavioral tests. All the experiments were conducted in a blind manner.

### BBB leakage examination in vivo

Evans blue extravasation was performed to investigate BBB permeability at 24 and 72 h after ICH as described previously^25^. In brief, Evans blue dye (2%; 4 ml/kg, Sigma-Aldrich) was infused >2 min into blood circulation through the left femoral vein. After 2 h, mice were euthanized via perfusion with 50 ml PBS through the left ventricle under anesthesia. Then, brains were removed and divided into left and right cerebral hemispheres for homogenate preparation. Each sample was weighed and homogenized in saline solution. After centrifugation (15,000*g*, 30 min), an equal volume of trichloroacetic acid was added to the resultant supernatant. The samples were centrifuged at 15,000*g* for 30 min after incubation overnight at 4 °C, and absorbance was quantified at 615 nm by a spectrophotometer.

Morphology of Evans blue leakage was observed on the slides using a confocal microscope (Zeiss, LSM780) equipped with a 633-nm HeNe laser as previously described^25^. In brief, the administration of Evans blue was the same as described above, the mice (*n* = 6, per group) were perfused with PBS and brains were removed. Frozen brain slices were prepared for coronal brain sections (30 μm) for observation after fixation in 4% paraformaldehyde at 4 °C for 24 h. ImageJ was used to measure the relative fluorescence intensity of Evans blue extravasation.

Moreover, FITC-dextran (70 kDa; Sigma-Aldrich) was perfused to visualize the vascular structure in the cortex and striatum of MEC17^fl/fl^ and Cdh5 Cre^ERT^:MEC17^fl/fl^ mice.

### Hematoma volume detection

Three days after ICH, the surviving mice in each group were imaged by a Bruker Biospec 7.0 T small-animal magnetic resonance imaging (MRI) scanner, with ADVANCE III hardware and software. Hematoma volume was measured by T2-weighted images and calculated using ImageJ software. Standard analysis methods were used to compute the images.

### Brain water content

At 24 and 72 h post-cerebral hemorrhage model creation, mice were anesthetized with a 2% mixture of isoflurane and air (1–2 l/min).The whole brain tissue was rapidly obtained, and the olfactory bulbs, cerebellum and brainstem were removed. Surface blood and water were absorbed with filter paper. Brain tissue was divided into left and right hemispheres, placed into preweighed Eppendorf tubes and weighed on an analytical balance (accuracy of 0.1 mg) to determine wet weight, then dried in an 80 °C incubator for 48 h to constant weight (error within 0.2 mg). After weighing the dry weight, the brain water content was calculated using the Elliot formula: brain water content = ((wet weight − dry weight)/wet weight) × 100%.

### RhoA-GTP assay

HBMECs were washed on ice with cold PBS and homogenized to ice-cold lysis buffer. The active RhoA was assessed utilizing a pull-down assay kit (#BK036S, Cytoskeleton) in accordance with the manufacturer’s instructions. The quantification of RhoA-GTP was performed through western blot (WB) analysis using an anti-RhoA antibody.

### Quantification for immunofluorescence staining and immunoblotting

To quantify and compare the mean fluorescence intensity (MFI) of MEC17, α-Ac-Tub, F-actin, NG2 and GFAP along the vessel, we first outlined the vessel’s contour and designated it as the region of interest (ROI). To quantify and compare the MFI of Evans blue extravasation in vivo, as well as Claudin5, ZO-1 and F-actin in cultured HBMECs, we similarly defined the field of view as the ROI. Densitometric measurements were then obtained using ImageJ software, after background subthresholding and area normalization. The number of IBA1- and GFAP-positive cells surrounding the hematoma was quantified and compared per field. All images used for analysis under multiple conditions were captured using the same optical parameters to avoid saturation. Stained tissue specimens were calculated using ImageJ in five randomly selected microscopic fields.

To analyze the band intensity values obtained from immunoblotting, we used a systematic approach utilizing ImageJ software for quantification. Specific ROIs were defined to accurately measure the intensity of each band as well as the background. The net intensity of each band was calculated by subtracting the background density from the band density. To account for variations in protein loading and transfer efficiency, the net intensity of each target band was normalized to the intensity of α-tubulin or β-actin. Finally, the control or sham group was assigned a value of 1, and the normalized intensity values of the other experimental groups were calculated relative to this control group. The normalized values were subsequently subjected to statistical analysis to assess significance between groups.

### Statistical analysis

Statistical analysis was performed using SPSS 18.0 software and GraphPad Prism 8.0 software. Data were expressed as the mean ± s.e.m. Comparisons between two groups were analyzed using two-tailed Student’s *t*-tests. Behavioral data collected at repeating time points were analyzed using two-way repeated-measures analysis of variance (ANOVA), followed by the Tukey’s post-hoc test. Other data were analyzed using one-way or two-way ANOVA followed by Tukey’s post-hoc test. A *P* value <0.05 was considered statistically significant.

## Results

### The expression of α-Ac-Tub and MEC17 was significantly decreased in ECs after ICH

**Fig. 1:**
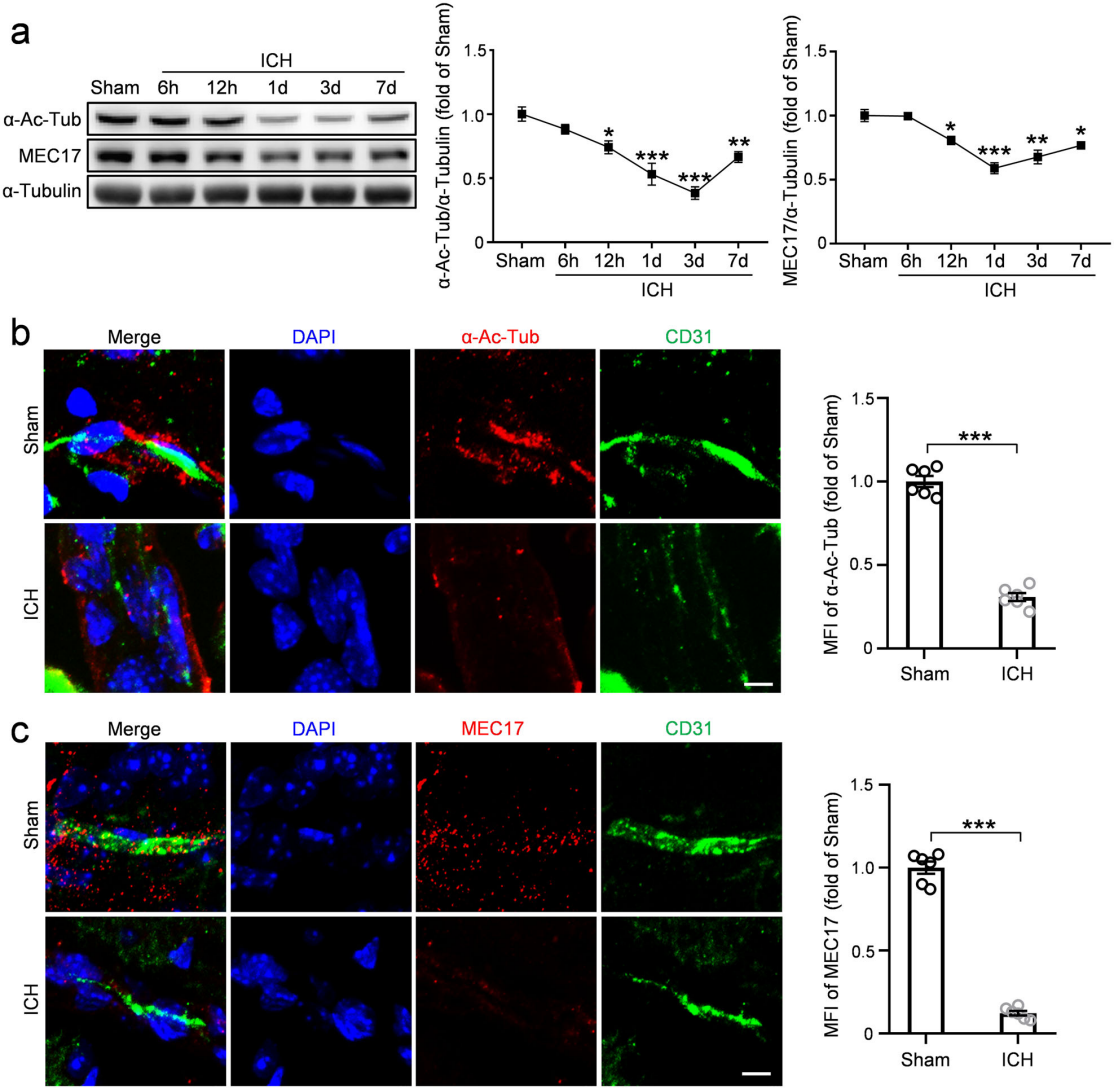
Changes in the expression of α-Ac-Tub and MEC17 after ICH. **a** The expression of α-Ac-Tub and MEC17 was evaluated in tissues around hematoma by WB at 6 h, 12 h, day 1, day 3 and day 7 after ICH. α-Tubulin was used as an internal loading control. Blots of α-Ac-Tub and MEC17 were quantified and expressed relative to the sham group (*n* = 4 animals per group). **b** Representative immunofluorescence pictures and statistical analysis of α-Ac-Tub (red) and the endothelial marker CD31 (green) in each group at day 3 after ICH. MFI of α-Ac-Tub was quantified and expressed relative to the sham group (*n* = 6 animals per group). Scale bar, 5 μm. **c** Representative immunofluorescence pictures and statistical analysis of MEC17 (red) and CD31 (green) in each group at day 3 after ICH. MFI of MEC17 was quantified and expressed relative to the sham group (*n* = 6 animals per group). Scale bar, 5 μm. Data are shown as the mean ± s.e.m. ***P < 0.001. One-way ANOVA followed by the Tukey’s post-hoc test for **a**, and two-tailed Student’s *t*-tests for **b** and **c**.

To investigate the role of α-Ac-Tub, a marker of stable microtubules, in maintaining EC structure and BBB function, we examined α-Ac-Tub levels in perihematomal tissues 6, 12 and 24 h and 3 and 7 days after ICH (Fig. 1a). WB analysis showed a significant decrease in α-Ac-Tub expression starting at 12 h after ICH, reaching its lowest point at day 3 (*P* < 0.001 versus sham group), followed by a slight recovery by day 7 (Fig. 1a).

Consistently, immunofluorescence staining revealed a significant reduction in MFI of α-Ac-Tub- and CD31-positive ECs along the vessels around the hematoma at day 3 after ICH compared with the sham group (Fig. 1b, *P* < 0.001; Supplementary Fig. 2a,b, *P* < 0.001). To evaluate the damage to the neurovascular unit, we also analyzed the MFI levels of two additional critical components of the BBB surrounding the vessels. NG2-positive pericytes exhibited a marked reduction (Supplementary Fig. 2a,c, *P* < 0.01), while GFAP-positive astrocytes demonstrated an increase in expression (Supplementary Fig. 2d,e, *P* < 0.05), indicative of astroglial scar formation. Because microtubule acetylation is mediated by MEC17, an acetyltransferase that specifically acetylates α-tubulin at the K40 site^13,26^, we went further to examine impacts of ICH on MEC17. Both WB and immunofluorescence analysis revealed significant reductions in MEC17 compared with the Sham group (Fig. 1a, c). These findings indicate a substantial decrease in both α-Ac-Tub and MEC17 in perihematomal ECs.

### Conditional knockout of MEC17 in ECs exacerbates BBB leakage, brain edema, inflammation, hematoma volume and motor dysfunction after ICH

Our previous study showed that MEC17 knockout reduces α-Ac-Tub levels by approximately 85% (ref. ^27^). To explore the link between reduced α-Ac-Tub in ECs and BBB leakage, we crossed endothelial-specific Cdh5 Cre^ERT^ mice^28^ with MEC17^fl/fl^ mice to conditionally knock out MEC17 in ECs (Cdh5 Cre^ERT^:MEC17^fl/fl^). Post-knockout analysis showed a significant reduction of MEC17 and α-Ac-Tub in CD31-positive vessels (Supplementary Fig. 3a, *P* < 0.001). WB of isolated ECs confirmed decreases in MEC17 and α-Ac-Tub to 16.1% and 24.2%, respectively (Supplementary Fig. 3b, *P* < 0.001). The residual MEC17 may be due to limited purity of the extracted ECs. Vascular structure visualization using dextran-FITC and BBB leakage assessment with Evans blue showed no significant differences between the Cdh5 Cre^ERT^:MEC17^fl/fl^ group and the MEC17^fl/fl^ group under normal conditions (Supplementary Fig. 3c–e, *P* > 0.05). These results confirmed that MEC17 was successfully ablated in ECs without affecting BBB function in normal conditions.

**Fig. 2:**
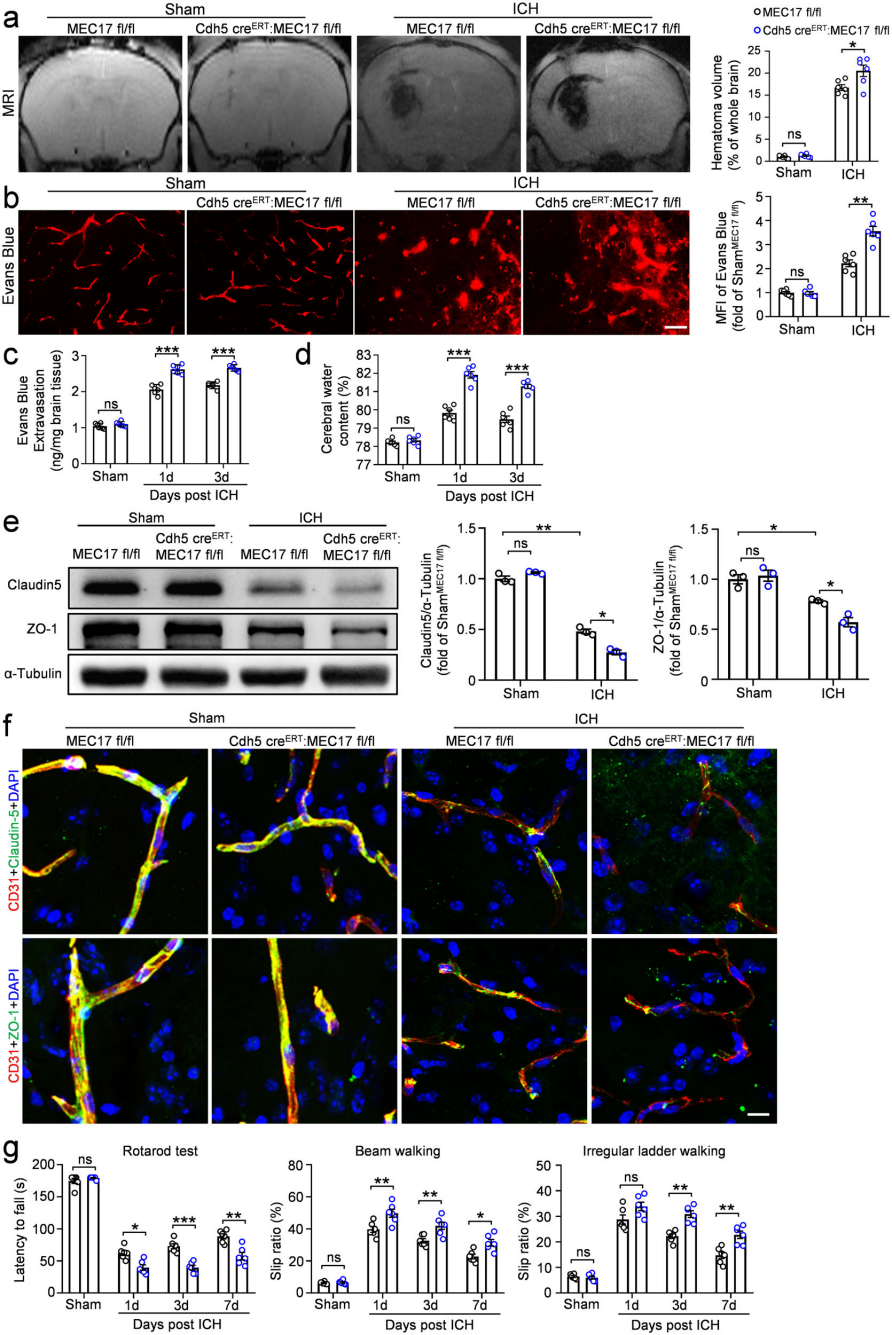
Conditional knockout of MEC17 in ECs aggravates BBB leakage, hematoma volume and motor dysfunction. **a** Representative MRI images showing the location and volume of hematoma in each group at day 3 after ICH. Hematoma volume was quantified and expressed as a percentage of the whole brain volume (*n* = 6 animals per group). **b** Representative Evans blue leakage images in each group at day 3 after ICH. MFI of Evans blue around hematoma was quantified and expressed relative to the sham^MEC17fl/fl^ group (*n* = 6 animals per group). Scale bar, 10 μm. **c** Evans blue extravasation around hematoma was measured using spectrophotometer in each group at days 1 and 3 after ICH (*n* = 6 animals per group). **d** The water content of the ipsilateral half-brain was measured in each group at days 1 and 3 after ICH (*n* = 6 animals per group). **e** The expression of Claudin5 and ZO-1 was evaluated in tissues around hematoma by WB at day 3 after ICH. α-Tubulin was used as an internal loading control. Blots of Claudin5 and ZO-1 were quantified and expressed relative to the sham^MEC17fl/fl^ group (*n* = 3 animals per group). **f** Representative immunofluorescence pictures of Claudin5 (green, top), ZO-1 (green, bottom) and the endothelial marker CD31 (red) in each group at day 3 after ICH (*n* = 3 animals per group). **g** Latency to fall on the rotarod, slip ratio of contralateral limbs on beam walking and irregular ladder walking were measured in each group at days 1, 3 and 7 after ICH (*n* = 6 animals per group). Data are shown as the mean ± s.e.m. *P < 0.05, **P < 0.01, ***P < 0.001. ns, not significant. Two-way ANOVA followed by the Tukey’s post-hoc test for **a**–**e**. Two-way repeated-measures ANOVA followed by the Tukey’s post-hoc test for **g**.

After ICH, MRI analysis revealed significantly larger hematoma volumes in Cdh5 Cre^ERT^:MEC17^fl/fl^ mice compared with the MEC17^fl/fl^ mice (Fig. 2a, *P* < 0.05). To further assess BBB disruption, we examined Evans blue extravasation from the peripheral circulation into the brain. Compared with MEC17^fl/fl^ mice, Cdh5 Cre^ERT^:MEC17^fl/fl^ mice exhibiting significantly greater Evans blue extravasation revealed worsened BBB disruption in Cdh5 Cre^ERT^:MEC17^fl/fl^ mice (Fig. 2b,c, *P* < 0.01), leading to more severe brain edema, as shown by increased water content (Fig. 2d, *P* < 0.001).

In the BBB, tight junction proteins between ECs are essential for maintaining barrier integrity and regulating tissue homeostasis^4,29^. To investigate the impact of ICH on these proteins, we analyzed the expression of Claudin5 and ZO-1 in perihematomal tissues at day 3 after ICH. WB results revealed significant reductions in both proteins after ICH (Fig. 2e, *P* < 0.01 for Claudin5, *P* < 0.05 for ZO-1), with further degradation observed in Cdh5 Cre^ERT^:MEC17^fl/fl^ mice (Fig. 2e, *P* < 0.05 for Claudin5 and ZO-1). Consistently, immunostaining showed a significant reduction in Claudin5 and ZO-1 expression after ICH, although the majority remained co-localized with CD31-positive ECs (Fig. 2f). In animals with MEC17 knockout in ECs, however, Claudin5 and ZO-1 expression was further decreased, appearing as punctate structures with minimal co-localization with ECs (Fig. 2f). These findings suggested that MEC17 in ECs not only regulates the expression of tight junction proteins but also influences their spatial distribution along ECs. In addition, knockout mice also exhibited more pronounced inflammatory responses compared with the MEC17^fl/fl^ mice, with increased microglia and astrocyte activation (Supplementary Fig. 4a, *P* < 0.05 for microglia, *P* < 0.01 for astrocytes).

Finally, to determine whether MEC17 deficiency in ECs affects neurological function in ICH mice, we assessed motor performance using the rotarod test, beam walking test and irregular ladder walking test at days 1, 3 and 7 after ICH. Compared with the MEC17^fl/fl^ group, deficiency of MEC17 in ECs led to exacerbated motor dysfunction, as indicated by a shortened latency to fall in the rotarod test (Fig. 2g; day 1, *P* < 0.01; day 3, *P* < 0.001; day 7, *P* < 0.01), an increased slip ratio of contralateral limbs both in beam walking test (Fig. 2g; day 1, *P* < 0.01; day 3, *P* < 0.01; day 7, *P* < 0.05) and an irregular ladder walking test (Fig. 2g; day 1, *P* > 0.05; day 3, *P* < 0.01; day 7, *P* < 0.01). These findings suggest that MEC17 and α-Ac-Tub deficiencies in ECs exacerbate BBB injury, brain edema, inflammation, hematoma volume and motor dysfunction after ICH, highlighting their potential as therapeutic targets for BBB protection.

### Overexpression of MEC17 mitigates BBB injury by increasing the expression of tight junction proteins

**Fig. 3:**
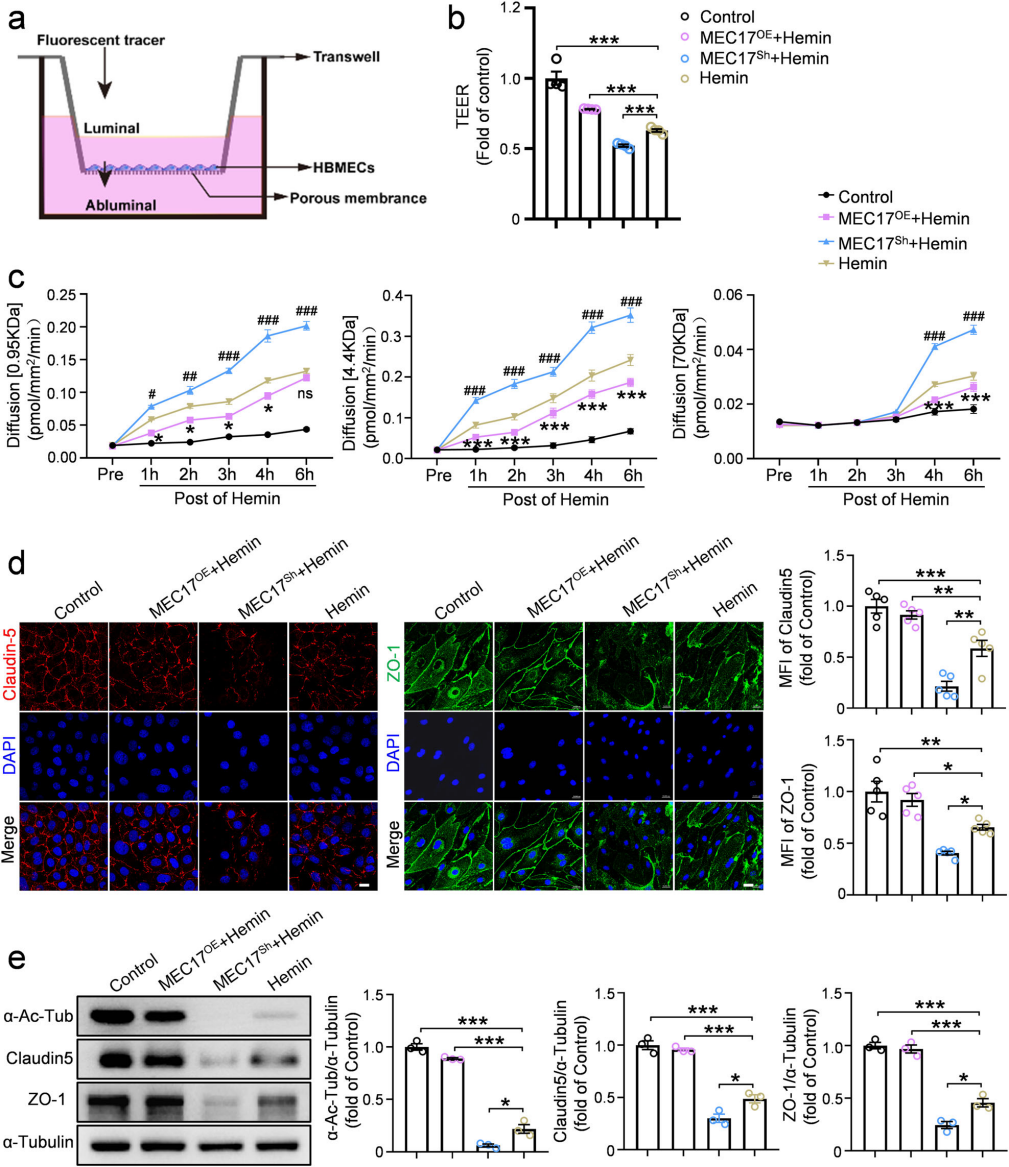
MEC17 overexpression alleviates barrier leakage and degradation of endothelial tight junction proteins after hemin treatment in an in vitro BBB model. **a** A schematic of the in vitro BBB model composed of HBMEC monolayers. **b** Quantification of TEER and expressed relative to the control of each group (*n* = 4 independent culture per group). **c** An HBMEC monolayer seeded on top of a membrane in the cell culture insert was subjected to hemin. The paracellular permeability was determined by measuring the luminal to abluminal diffusion rates of the 0.95-kDa Alexa 555 cadaverine (left), the 4.4-kDa TRITC-dextran (middle) or the 70-kDa FITC-dextran (right) before (Pre) and 1–6 h after hemin treatment (*n* = 4 independent culture per group). *P < 0.05, **P < 0.01, ***P < 0.001 for MEC17^OE^+hemin versus hemin. ^#^*P* < 0.05, ^##^*P* < 0.01, ^###^*P* < 0.001 for MEC17^Sh^+hemin versus hemin. **d** HBMECs were infected with control empty lentivirus (Control), or lentiviral vectors carrying MEC17 (MEC17^OE^) or lentiviral vectors carrying MEC17-shRNA (MEC17^Sh^). Synchronously, HBMECs were treated with vehicle or hemin. Representative immunofluorescence pictures and statistical analysis of Claudin5 (red, left) and the ZO-1 (green, right) in each group at 6 h after hemin treatment (*n* = 5 independent culture per group). The MFI of Claudin5 and ZO-1 was quantified and expressed relative to the control group. Scale bar, 20 μm. **e** The expression of α-Ac-Tub, ZO-1 and Claudin5 was evaluated in each group by WB at 6 h after hemin treatment. α-Tubulin was used as an internal loading control. Blots of α-Ac-Tub, ZO-1 and Claudin5 were quantified and expressed relative to the control group (*n* = 3 independent culture per group). Data are shown as the mean ± s.e.m. *P < 0.05, **P < 0.01, ***P < 0.001. ns, not significant. One-way ANOVA followed by the Tukey’s post-hoc test for **a**, **d** and **e**. Two-way repeated-measures ANOVA followed by the Tukey’s post-hoc test for **c**.

To further investigate the mechanisms underlying ICH-induced BBB disruption, we adopted an in vitro BBB model using a monolayer of HBMECs (Fig. 3a). The expression of MEC17 in HBMECs was either increased or decreased with lentivirus-mediated manipulations (Supplementary Fig. 5a, *P* < 0.001 for MEC17^Sh^ versus control, *P* < 0.01 for MEC17^OE^ versus control). Correspondingly, the MFI of α-Ac-Tub was significantly reduced in the MEC17^Sh^ group (Supplementary Fig. 5b, *P* < 0.001 for MEC17^Sh^ versus control) and increased in the MEC17^OE^ group (Supplementary Fig. 5b, *P* < 0.01 for MEC17^OE^ versus control).

After validating the model, we administrated a 24-h hemin treatment to mimic ICH conditions and evaluated its impact on barrier integrity and permeability. Hemin significantly reduced TEER (Fig. 3b, *P* < 0.001), one of the most widely used indicators to quantify the barrier integrity of endothelial layers^30,31^. Silencing of MEC17 exacerbated the injury of TEER (Fig. 3b, *P* < 0.001), while overexpression of MEC17 alleviated the injury of TEER (Fig. 3b, *P* < 0.001). We then assessed luminal-to-abluminal permeability using fluorescent tracers of varying molecular weights. Compared with non-hemin-treated cultures, hemin induced a progressive increase in barrier permeability, as indicated by increased diffusion rates. In particular, the two small-size tracers (0.95 kDa cadaverine and 4.4 kDa dextran) crossed the EC barrier within 1 h after hemin treatment, whereas the larger tracer (70 kDa dextran) remained restricted for the first 3 h but crossed by 4–6 h (Fig. 3c). MEC17 overexpression significantly reduced, but did not entirely prevent, the leakage of small tracers (1–6 h) and the large tracer (4–6 h), whereas MEC17 silencing further aggravated barrier permeability (Fig. 3c). These findings revealed that endothelial MEC17 and α-Ac-Tub play a critical role in attenuating hemin-induced BBB leakage to both small- and large-size macromolecules in vitro.

To confirm this further, we performed immunofluorescence staining and WB to assess Claudin5 and ZO-1 expression in the HBMEC monolayer. Compared with the control group, hemin treatment significantly reduced the expression of Claudin5 (Fig. 3d,e, *P* < 0.001) and ZO-1 (Fig. 3d, *P* < 0.01; Fig. 3e, *P* < 0.001). Overexpression of MEC17 mitigated hemin-induced tight junction degradation, significantly preserving Claudin5 (Fig. 3d, *P* < 0.01; Fig. 3e, *P* < 0.001) and ZO-1 (Fig. 3d, *P* < 0.05; Fig. 3e, *P* < 0.001) expression. Conversely, MEC17 silencing exacerbated tight junction protein loss, further reducing Claudin5 (Fig. 3d, *P* < 0.01; Fig. 3e, *P* < 0.05) and ZO-1 (Fig. 3d, *P* < 0.05; Fig. 3e, *P* < 0.05) levels compared with the hemin-treated group. Thus, MEC17 overexpression protects against hemin-induced barrier injury by preserving tight junction protein expression.

### Conditional overexpression of MEC17 in ECs using AAV-BI30 effectively alleviates BBB leakage, brain edema, inflammation, hematoma volume and motor dysfunction after ICH

For potential clinical translation, we next used AAV-BI30 for gene manipulations, which efficiently labels brain microvascular ECs in mouse via intravenous injection^32^. Our findings demonstrated an approximately 80% co-localization rate of AAV-BI30-GFP with laminin-labeled microvessels 3 weeks after injection (Supplementary Fig. 6a,b). In addition, injection of AAV-BI30-MEC17-GFP significantly increased MEC17 expression compared with the AAV-BI30-GFP group (Supplementary Fig. 6c,d, *P* < 0.05).

**Fig. 4:**
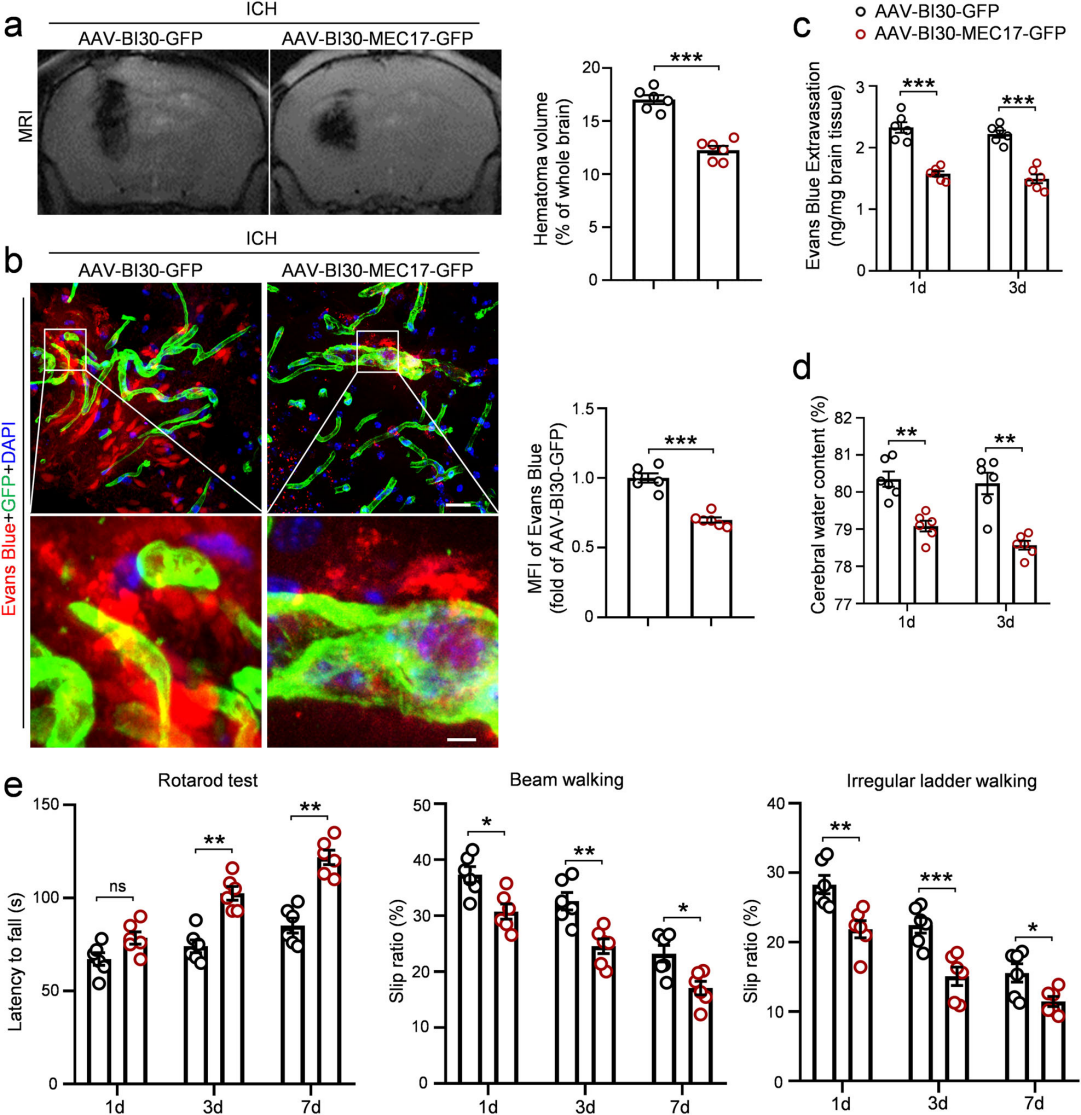
MEC17 overexpression alleviates BBB leakage, hematoma volume and motor dysfunction after ICH. **a** Representative MRI images to show the location and volume of hematoma in each group at day 3 after ICH. Hematoma volume was quantified and expressed as percentage of the whole brain volume (*n* = 6 animals per group). **b** Representative Evans blue leakage and EC-GFP images in each group at day 3 after ICH. MFI of Evans Blue around hematoma was quantified and expressed relative to the AAV-BI30-GFP group (n = 6 animals per group). Scale bar, 20 μm (top) and 50 μm (bottom). **c** Evans blue extravasation around hematoma was measured using spectrophotometer in each group at days 1 and 3 after ICH (*n* = 6 animals per group). **d** The water content of the ipsilateral half-brain was measured in each group at days 1 and 3 after ICH (*n* = 6 animals per group). **e** Latency to fall on the rotarod, slip ratio of contralateral limbs on beam walking and irregular ladder walking were measured in each group at days 1, 3 and 7 after ICH (*n* = 6 animals per group). Data are shown as the mean ± s.e.m. *P < 0.05, **P < 0.01, ***P < 0.001. ns, not significant. Two-tailed Student’s *t*-tests for **a**–**d**. Two-way repeated-measures ANOVA followed by the Tukey’s post-hoc test for **e**.

We next examined the therapeutic effects of MEC17 overexpression in ECs for treating ICH. MRI results revealed a significant reduction in hematoma volume in AAV-BI30-MEC17-GFP mice compared with AAV-BI30-GFP mice (Fig. 4a, *P* < 0.001). The MFI of Evans blue in perilesional tissue was also lower in AAV-BI30-MEC17-GFP mice (Fig. 4b, *P* < 0.001). Furthermore, compared with AAV-BI30-GFP mice, Evans blue extravasation (Fig. 4c, *P* < 0.001) and brain water content (Fig. 4d, *P* < 0.01) in perihematomal tissues significantly decreased at days 1 and 3 after ICH in AAV-BI30-MEC17-GFP mice. Overexpression of MEC17 also reduced the number of activated microglia and astrocytes in perihematomal tissues (Supplementary Fig. 4b, *P* < 0.05 for microglia, *P* < 0.001 for astrocytes).

Finally, MEC17 overexpression in ECs lengthened the latency to fall in the rotarod test (Fig. 4e; day 1, *P* > 0.05; day 3, *P* < 0.01; day 7, *P* < 0.01) and reduced the slip ratio of contralateral limbs in both the beam walking test (Fig. 4e; day 1, *P* < 0.05; day 3, *P* < 0.01; day 7, *P* < 0.05) and the irregular ladder walking test (Fig. 4e; day 1, *P* < 0.01; day 3, *P* < 0.001; day 7, *P* < 0.05). Collectively, these findings indicate that MEC17 overexpression in ECs mitigates BBB injury, brain edema, inflammation, hematoma volume and motor dysfunction after ICH.

### TBA intervention attenuated BBB disruption and motor dysfunction by preventing tight junction protein degradation

To further investigate whether α-Ac-Tub mediates MEC17-induced protection of ECs, we used TBA (an HDAC6 inhibitor), the deacetylase responsible for modulating α-Ac-Tub levels^20,33^.

**Fig. 5:**
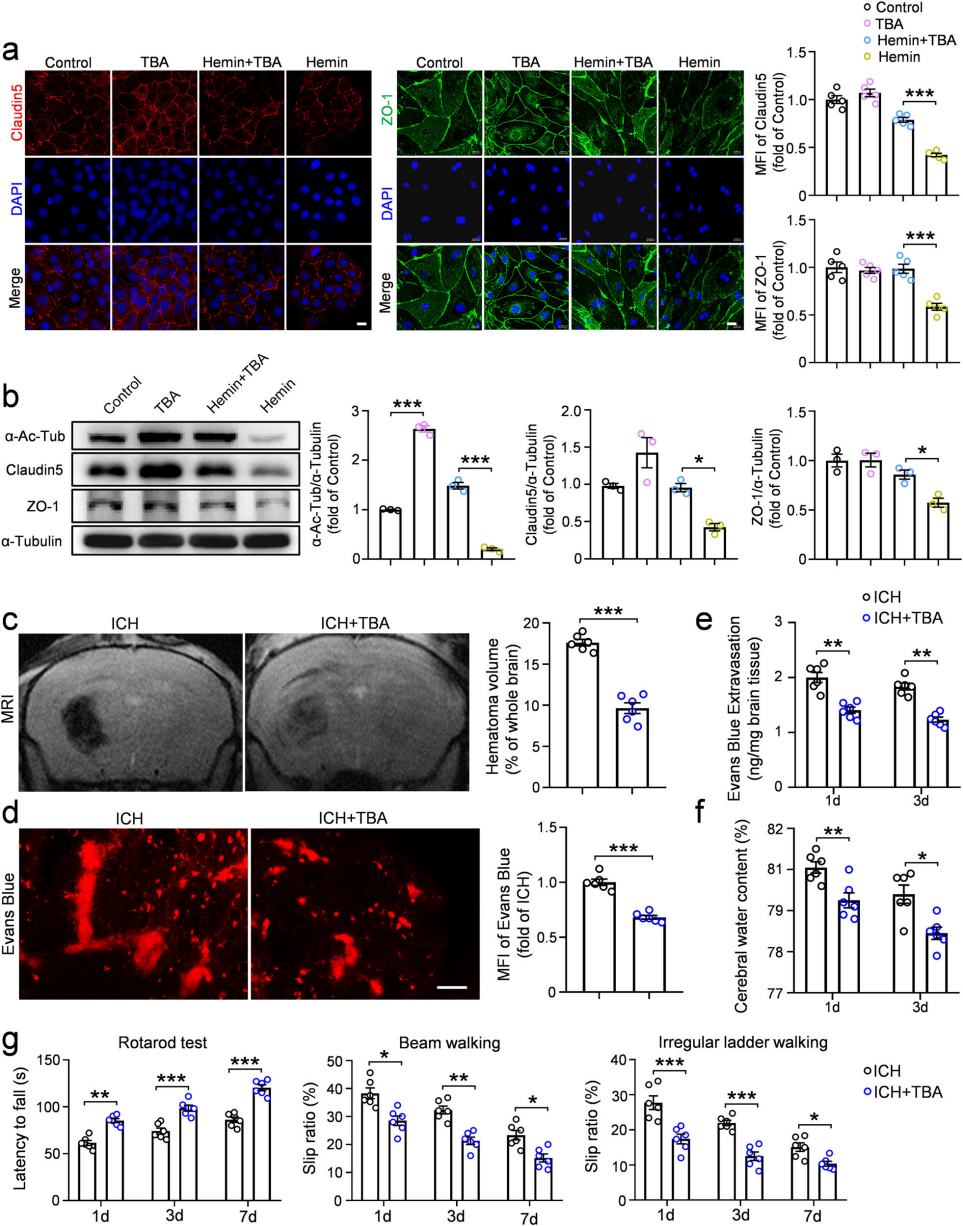
TBA treatment alleviates barrier injury in vivo and in vitro, decreases hematoma volume and improves motor function after ICH. **a** Representative immunofluorescence pictures and statistical analysis of Claudin5 (red, left) and the ZO-1 (green, right) in each group at 6 h after hemin treatment. The MFI of Claudin5 and ZO-1 was quantified and expressed relative to the control group (*n* = 5 independent culture per group). Scale bar, 20 μm. **b** The expression of α-Ac-Tub, Claudin5 and ZO-1 was evaluated in each group by WB at 6 h after hemin treatment. α-Tubulin was used as an internal loading control. Blots of α-Ac-Tub, Claudin5 and ZO-1 were quantified and expressed relative to the control group (*n* = 3 independent culture per group). **c** Representative MRI images to show the location and volume of hematoma in each group at day 3 after ICH. Hematoma volume was quantified and expressed as percentage of the whole brain volume (*n* = 6 animals per group). **d** Representative Evans blue leakage images in each group at day 3 after ICH. MFI of Evans blue around hematoma was quantified and expressed relative to the ICH group (*n* = 6 animals per group). Scale bar, 10 μm. **e** Evans blue extravasation around hematoma was measured using a spectrophotometer in each group at days 1 and 3 after ICH (*n* = 6 animals per group). **f** The water content of the ipsilateral half-brain was measured in each group at days 1 and 3 after ICH (*n* = 6 animals per group). **g** Latency to fall on the rotarod, slip ratio of contralateral limbs on beam walking and irregular ladder walking were measured in each group at days 1, 3 and 7 after ICH (*n* = 6 animals per group). Data are shown as the mean ± s.e.m. *P < 0.05, **P < 0.01, ***P < 0.001. One-way ANOVA followed by the Tukey’s post-hoc test for **a** and **b**. Two-tailed Student’s *t*-tests for **c**–**f**. Two-way repeated-measures ANOVA followed by the Tukey’s post-hoc test for **g**.

In vitro, immunofluorescence and WB analyses showed that TBA treatment significantly increased α-Ac-Tub expression (Fig. 5a,b, *P* < 0.001) and mitigated hemin-induced degradation of Claudin5 and ZO-1 (Fig. 5a,b) compared with the control group.

In vivo, MRI analysis showed a significant reduction in hematoma volume in the ICH + TBA group compared with the ICH group (Fig. 5c, *P* < 0.001). The MFI of Evans blue in the perilesional tissue (Fig. 5d, *P* < 0.001), Evans blue extravasation (Fig. 5e, *P* < 0.01) and brain water content (Fig. 5f, *P* < 0.01 for day 1, *P* < 0.05 for day 3) in perihematomal tissues were significantly decreased in the ICH + TBA group. Furthermore, TBA treatment significantly reduced the number of activated microglia and astrocytes in perihematomal tissues (Supplementary Fig. 4b, *P* < 0.01 for microglia, *P* < 0.001 for astrocytes). As a result, motor function was significantly improved in ICH mice treated with TBA. Compared with the ICH group, TBA treatment increased the latency to fall in the rotarod test (Fig. 5g; day 1, *P* < 0.01; day 3, *P* < 0.001; day 7, *P* < 0.001) and decreased the slip ratio of contralateral limbs in both the beam walking test (Fig. 5g; day 1, *P* < 0.05; day 3, *P* < 0.01; day 7, *P* < 0.05) and the irregular ladder walking test (Fig. 5g; day 1, *P* < 0.001; day 3, *P* < 0.001; day 7, *P* < 0.05). Mechanistically, TBA treatment significantly rescued the expression of α-Ac-Tub and CD31 along the vessels but had minimal impact on the surrounding NG2-positive pericytes and GFAP-positive astrocytes (Supplementary Fig. 2a–e). Together, these findings suggest that the TBA-induced increase in α-Ac-Tub effectively reduces BBB leakage, brain edema, inflammation, hematoma volume and motor dysfunction by preventing the degradation of tight junction proteins after ICH.

### α-Ac-Tub protects BBB function by preventing stress fiber formation

**Fig. 6:**
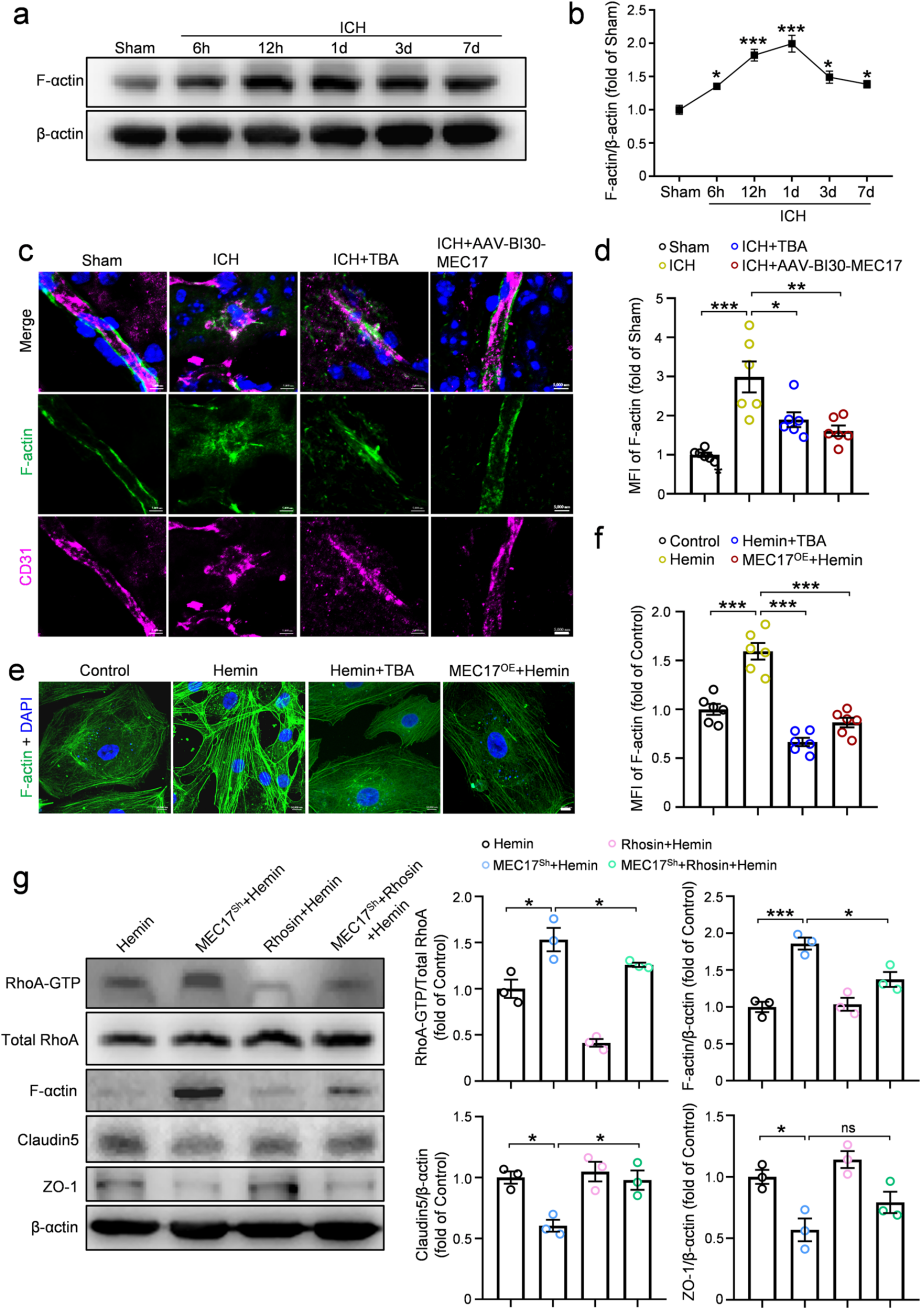
α-Ac-Tub alleviates BBB injury through inhibiting the stress fiber formation. **a** The expression of F-actin was evaluated in tissues around hematoma by WB at 6 h, 12 h, 1 day, 3 days and 7 days after ICH. β-Actin was used as an internal loading control. **b** The blot of F-actin was quantified and expressed relative to the sham group (*n* = 3 animals per group). **c** Representative immunofluorescence pictures of F-actin (green) and CD31 (magenta) in each group at day 3 after ICH. Scale bar, 5 μm. **d** The MFI of F-actin was quantified and expressed relative to the sham group (*n* = 6 animals per group). **e**, HBMECs were treated with vehicle, hemin or hemin+TBA or infected with lentiviral vectors carrying MEC17 (MEC17^OE^). Representative immunofluorescence pictures of F-actin in each group at 6 h after hemin treatment. Scale bar, 10 μm. **f** The MFI of F-actin was quantified and expressed relative to the control group (*n* = 6 independent culture per group). **g** The expression of RhoA-GTP, RhoA, F-actin, Claudin5 and ZO-1 was evaluated in each group by WB at 24 h after hemin treatment. Total RhoA was used as an internal loading control of RhoA-GTP; β-actin was used as an internal loading control for other proteins. Blots of RhoA-GTP, F-actin, Claudin5 and ZO-1 were quantified and expressed relative to the hemin group (*n* = 3 independent culture per group). Data are shown as the mean ± s.e.m. **P < 0.01, ***P < 0.001. One-way ANOVA followed by the Tukey’s post-hoc test.

Aberrant actin polymerization, forming stress fibers (F-actin), is known to rapidly occur after cerebral ischemia, contributing to structural damage in ECs and BBB disruption^6,7^. To assess F-actin dynamics after ICH, we analyzed its expression in perihematomal tissues at multiple time points using WB (Fig. 6a). Our results showed that its expression increased significantly from 6 h, peaking at day 1 after ICH (Fig. 6b; 6 h, *P* < 0.05 versus sham; 12 h, *P* < 0.001 versus sham; day 1, *P* < 0.001 versus sham; day 3 and 7, *P* < 0.05 versus sham). Morphologically, robust F-actin expression was detected in CD31-positive ECs around the hematoma (Fig. 6c,d, *P* < 0.001) and in HBMECs after hemin treatment (Fig. 6e,f, *P* < 0.001). These results confirmed that F-actin expression rapidly increases in ECs after ICH.

Previous studies have shown that α-Ac-Tub overexpression inhibits TNF-induced microtubule disassembly and subsequent actin stress fiber formation in ECs after pulmonary edema^34,35^. However, the relationship between α-Ac-Tub and F-actin after ICH remains unclear. To investigate this, we first examined F-actin expression in MEC17^Sh^ and MEC17^OE^ HBMECs. Compared with the control group, F-actin MFI was significantly increased in the MEC17^Sh^ group (Supplementary Fig. 5b, *P* < 0.01 for MEC17^Sh^ versus control) and decreased in the MEC17^OE^ group (Supplementary Fig. 5b, *P* < 0.001 for MEC17^OE^ versus control), indicating an inverse relationship between α-Ac-Tub and F-actin. Next, we examined F-actin expression after TBA and AAV-BI30-MEC17-GFP treatments in both in vivo and in vitro models. Interestingly, both TBA and AAV-BI30-MEC17 significantly reduced F-actin expression in CD31-positive ECs around the hematoma after ICH compared with the ICH group (Fig. 6c,d, *P* < 0.05 for ICH + TBA versus ICH, *P* < 0.01 for ICH + AAV-BI30-MEC17 versus ICH). Similarly, F-actin expression was significantly decreased in HBMECs treated with TBA or MEC17^OE^ after hemin exposure compared with the hemin group (Fig. 6e,f, *P* < 0.001). These findings suggest that α-Ac-Tub plays a key role in suppressing actin stress fiber formation after ICH, thereby potentially contributing to BBB protection.

Previous studies have shown that microtubule disassembly promotes actin cytoskeletal remodeling and increases EC permeability by releasing microtubule-bound Rho GTPase-specific guanine nucleotide exchange factors (GEFs)^36,37^. To investigate how α-Ac-Tub regulates F-actin stress fibers, we used rhosin, a specific RhoA inhibitor that blocks RhoA-GEF interactions^38^. Our results demonstrated that MEC17 silencing exacerbated the hemin-induced elevation of RhoA-GTP (Fig. 6g, *P* < 0.05) and F-actin (Fig. 6g, *P* < 0.001) in cultured HBMECs, accompanied by a further reduction in Claudin5 and ZO-1 expression (Fig. 6g, *P* < 0.05). Notably, rhosin treatment partially reversed the increases in RhoA-GTP and F-actin levels induced by of MEC17 silencing with hemin treatment, and restored the expression of Claudin5 (Fig. 6g, *P* < 0.05), but not that of ZO-1 (Fig. 6g, *P* > 0.05). Taken together, those findings indicate that α-Ac-Tub protects BBB function by preventing stress fiber formation via a RhoA-dependent pathway.

## Discussion

This study characterizes early BBB disruption in an experimental ICH model, elucidates the underlying mechanisms and investigates the role of α-Ac-Tub in the injury of ECs. The major findings of the present study include the following. (1) We identified a remarkable decrease in α-Ac-Tub, a marker of stable microtubules, in ECs after ICH. Conditional knockout of MEC17 in ECs further suppressed α-Ac-Tub expression, exacerbating BBB leakage. Conversely, overexpression of MEC17 via AAV-BI30-MEC17 increased α-Ac-Tub levels, reducing BBB leakage and brain injury by enhancing tight junction protein expression. (2) Inhibition of HDAC6 with TBA, a deacetylase of α-Ac-Tub, mitigated BBB leakage, reduced secondary brain injury and improved neurological recovery. (3) Mechanistically, α-Ac-Tub protects BBB function by preventing actin stress fiber formation via a RhoA-dependent pathway.

BBB destruction is a pivotal contributor to secondary brain injuries after ICH, leading to brain edema, inflammatory responses, neuronal death and neurological deficits. As ECs serve as the primary defense of the BBB, understanding the regulation of their permeability under pathological conditions is critical for identifying the mechanisms of microvascular dysfunction and developing clinical prevention strategies. The structural integrity of the endothelial barrier is maintained through a complex interplay of cytoskeletal elements, cell–substrate focal adhesions and adhesive cell junctions^39^. Changes in endothelial permeability, mediated by various agonists, are directly linked to the reorganization of the actomyosin cytoskeleton^35,40–44^. This process leads to cell contraction and the opening of intercellular gaps or the enhancement of the cortical actin cytoskeleton, strengthening the endothelial barrier. Recent studies highlight the essential role of microtubules in regulating endothelial permeability, influencing cytoskeletal organization, actomyosin contractility, paracellular gaps and stress fiber formation^45^. However, the impact of microtubules on EC injury after ICH remains largely unexplored and underestimated. In this study, we demonstrated that decreased expression of α-Ac-Tub, one marker of stable microtubules, aggravated BBB leakage by reducing tight junction proteins in vitro and in vivo. This reduction further exacerbated secondary brain injury, leading to increased hematoma size, brain edema, neuroinflammation and impaired neurological function. By contrast, pharmacological or genetic overexpression of α-Ac-Tub in ECs alleviated BBB damage and improved functional recovery after ICH, underscoring the crucial role of microtubule stability in maintaining EC and BBB integrity. In this regard, another study found that inhibitors of microtubule polymerization such as nocodazole directly increase endothelial permeability, an effect that can be prevented by pretreatment with the microtubule stabilizer paclitaxel^44^.

The microtubule cytoskeleton serves as a central framework for intracellular signaling, with its dynamic complexity and functional versatility driven primarily by posttranslational modifications such as acetylation, detyrosination and SUMOylation^46–48^. Acetylation of α-tubulin at Lys-40, a highly conserved modification, enhances the microtuble flexibility and durability. This process is mediated by MEC17, an acetyltransferase that specifically acetylates α-tubulin at the K40 site^13,26^. MEC17 deletion results in a significant reduction of α-Ac-Tub^49,50^, but whether it contributes to BBB damage via alternative pathways remains an open question. In this study, MEC17 knockout in ECs downregulated α-Ac-Tub, exacerbating BBB damage. Notably, TBA, an HDAC6 inhibitor^20,51^, restored α-Ac-Tub levels, reducing BBB leakage, minimizing secondary brain injury and improving neurological recovery, highlighting α-Ac-Tub’s critical role in BBB integrity after ICH.

AAV gene therapy has emerged as a focal point in gene therapy research, with its clinical applications progressively maturing. In this context, AAV-BI30, capable of efficiently and specifically labeling cerebral ECs^32^, has garnered our attention. Using AAV-BI30, we achieved targeted overexpression of MEC17 and α-Ac-Tub in ECs. This intervention mitigated BBB leakage, decreased secondary brain injury and promoted neurological recovery after ICH, aligning with the beneficial effects observed with TBA treatment. Interestingly, TBA demonstrated a more pronounced therapeutic effect compared with AAV-BI30-MEC17 treatment, possibly due to its broader impact on both ECs and surrounding neurons near the hematoma. Despite the promising therapeutic potential of MEC17, the absence of specific inhibitors has limited further exploration of its functions. However, a recent bioscreening has identified potential MEC17 inhibitors^52^, paving the way for further investigation into MEC17’s role and potential therapeutic applications.

While maintaining α-Ac-Tub in ECs reduces BBB damage, the exact regulatory mechanisms are not fully understood. Research has highlighted the importance of actin cytoskeleton remodeling in regulating the endothelial barrier following ICH. Recent studies have shown that actin cytoskeleton remodeling plays a key role in endothelial barrier regulation after ICH, with persistent actin polymerization driven by ROCK–MLC signaling contributing to early BBB disruption^7^. HSP27 prevents early BBB leakage induced by ischemia–reperfusion by inhibiting actin polymerization and junctional protein disassembly in cerebral ECs^6^. However, the role of microtubules in this process is less well explored. It appears that microtubule disassembly is an early event in thrombin-induced endothelial barrier disruption, leading to increased EC permeability and the release of microtubule-bound Rho GTPase-specific GEFs, which activate a Rho-dependent pathway for actin cytoskeletal remodeling^37^. GEF-H1, a Rho-specific GEF, is a crucial signaling molecule linking microtubules and actin cytoskeleton^36,53^. Mechanistically, Stathmin promotes microtubule depolymerization by dephosphorylation, which releases Rho-specific GEF-H1 from microtubules^54^. This, in turn, activates Rho, increases p-MLC levels and promotes stress fiber formation^54^. Nocodazole, a microtubule inhibitor, further supports the role of microtubules in maintaining endothelial integrity by demonstrating increased EC permeability upon treatment^55^.

Accumulating evidence suggested that hemin, a hemoglobin breakdown product, significantly contributes to endothelial barrier dysfunction after ICH by inducing actin polymerization and cytoskeletal reorganization^56–58^. Interestingly, our study demonstrated that reduced α-Ac-Tub levels in ECs after ICH and hemin treatment promote stress fiber formation and tight junction disassembly via a RhoA-dependent pathway. Targeting α-Ac-Tub with gene intervention and TBA treatment increased α-Ac-Tub expression in ECs, which helped to maintain the stability of the endothelial microtubule structure. These approaches inhibited stress fiber formation and tight junction disassembly, slowing the progression of EC damage and preserving vascular permeability.

This study has several limitations. First, while MEC17 and TBA interventions highlight the role of α-Ac-Tub in endothelial barrier integrity, we did not directly target the K40 site of α-Ac-Tub. Future studies should focus on this specific modification to provide more direct evidence of its functional significance. Second, although our findings suggest that RhoA mediates the effects of α-Ac-Tub on F-actin dynamics, RhoA may also regulate microtubule dynamics^59^, warranting further mechanistic exploration. Third, behavioral assessments were limited to 7 days; extending the observation period would provide deeper insights into the long-term effects of α-Ac-Tub on endothelial function and BBB integrity.

## Supplementary information

Supplementary Information 1

Supplementary Information 2

## Data Availability

Data will be made available on request.

## References

[1] Hu, R. et al. Long-term outcomes and risk factors related to hydrocephalus after intracerebral hemorrhage. *Transl. Stroke Res.* **12**, 31–38 (2021). 32514905 10.1007/s12975-020-00823-y

[2] Zhang, C. et al. Clot removAl with or without decompRessive craniectomy under ICP monitoring for supratentorial IntraCerebral Hemorrhage (CARICH): a randomized controlled trial. *Int J. Surg.* **110**, 4804–4809 (2024). 38640513 10.1097/JS9.0000000000001466 PMC11325930

[3] Fang, Y. et al. Deficiency of TREK-1 potassium channel exacerbates blood–brain barrier damage and neuroinflammation after intracerebral hemorrhage in mice. *J. Neuroinflammation* **16**, 96 (2019). 31072336 10.1186/s12974-019-1485-5 PMC6506965

[4] Zhao, Z. et al. Establishment and dysfunction of the blood–brain barrier. *Cell* **163**, 1064–1078 (2015). 26590417 10.1016/j.cell.2015.10.067 PMC4655822

[5] Storck, S. E., Kurtyka, M. & Pietrzik, C. U. Brain endothelial LRP1 maintains blood–brain barrier integrity. *Fluids Barriers CNS* **18**, 27 (2021). 34147102 10.1186/s12987-021-00260-5 PMC8214794

[6] Shi, Y. et al. Endothelium-targeted overexpression of heat shock protein 27 ameliorates blood–brain barrier disruption after ischemic brain injury. *Proc. Natl Acad. Sci. USA* **114**, E1243–E1252 (2017). 28137866 10.1073/pnas.1621174114 PMC5320958

[7] Shi, Y. et al. Rapid endothelial cytoskeletal reorganization enables early blood–brain barrier disruption and long-term ischaemic reperfusion brain injury. *Nat. Commun.* **7**, 10523 (2016). 26813496 10.1038/ncomms10523 PMC4737895

[8] Nakajima, H. & Mochizuki, N. Flow pattern-dependent endothelial cell responses through transcriptional regulation. *Cell Cycle* **16**, 1893–1901 (2017). 28820314 10.1080/15384101.2017.1364324 PMC5638382

[9] Cao, H. et al. Hypoxia destroys the microstructure of microtubules and causes dysfunction of endothelial cells via the PI3K/Stathmin1 pathway. *Cell Biosci.* **9**, 20 (2019). 30820314 10.1186/s13578-019-0283-1 PMC6380067

[10] Gorovoy, M. et al. LIM kinase 1 coordinates microtubule stability and actin polymerization in human endothelial cells. *J. Biol. Chem.* **280**, 26533–26542 (2005). 15897190 10.1074/jbc.M502921200 PMC1403832

[11] Shen, Y. & Ori-McKenney, K. M. Microtubule-associated protein MAP7 promotes tubulin posttranslational modifications and cargo transport to enable osmotic adaptation. *Dev Cell* **59**, 1553–1570.e7 (2024). 10.1016/j.devcel.2024.03.022 PMC11187767 38574732

[12] Li, L. & Yang, X. J. Tubulin acetylation: responsible enzymes, biological functions and human diseases. *Cell Mol. Life Sci.* **72**, 4237–4255 (2015). 26227334 10.1007/s00018-015-2000-5 PMC11113413

[13] Akella, J. S. et al. MEC-17 is an α-tubulin acetyltransferase. *Nature* **467**, 218–222 (2010). 20829795 10.1038/nature09324 PMC2938957

[14] Castro-Castro, A. et al. ATAT1/MEC-17 acetyltransferase and HDAC6 deacetylase control a balance of acetylation of α-tubulin and cortactin and regulate MT1-MMP trafficking and breast tumor cell invasion. *Eur. J. Cell Biol.* **91**, 950–960 (2012). 22902175 10.1016/j.ejcb.2012.07.001

[15] Portran, D. et al. Tubulin acetylation protects long-lived microtubules against mechanical ageing. *Nat. Cell Biol.* **19**, 391–398 (2017). 28250419 10.1038/ncb3481 PMC5376231

[16] Xu, Z. et al. Microtubules acquire resistance from mechanical breakage through intralumenal acetylation. *Science* **356**, 328–332 (2017). 28428427 10.1126/science.aai8764 PMC5457157

[17] Prasain, N. et al. Soluble adenylyl cyclase-dependent microtubule disassembly reveals a novel mechanism of endothelial cell retraction. *Am. J. Physiol. Lung Cell Mol. Physiol.* **297**, L73–L83 (2009). 19395666 10.1152/ajplung.90577.2008 PMC2711814

[18] Krafft, P. R. et al. Modeling intracerebral hemorrhage in mice: injection of autologous blood or bacterial collagenase. *J. Vis. Exp.* **67**, e4289 (2012). 10.3791/4289 PMC3490262 23023153

[19] Yang, W. et al. Exosomes from young healthy human plasma promote functional recovery from intracerebral hemorrhage via counteracting ferroptotic injury. *Bioact. Mater.* **27**, 1–14 (2023). 37006825 10.1016/j.bioactmat.2023.03.007 PMC10060149

[20] Wang, Z. et al. Tubastatin A, an HDAC6 inhibitor, alleviates stroke-induced brain infarction and functional deficits: potential roles of α-tubulin acetylation and FGF-21 up-regulation. *Sci. Rep.* **6**, 19626 (2016). 26790818 10.1038/srep19626 PMC4726180

[21] Yang, C. et al. Acetylated α-tubulin alleviates injury to the dendritic spines after ischemic stroke in mice. *CNS Neurosci. Ther.* **29**, 2327–2338 (2023). 36965035 10.1111/cns.14184 PMC10352872

[22] Imai, T. et al. Intracellular Fe^2+^ accumulation in endothelial cells and pericytes induces blood–brain barrier dysfunction in secondary brain injury after brain hemorrhage. *Sci. Rep.* **9**, 6228 (2019). 30996325 10.1038/s41598-019-42370-z PMC6470176

[23] Kushwaha, R. et al. Reactive astrocytes associated with prion disease impair the blood brain barrier. *Neurobiol. Dis.* **185**, 106264 (2023). 37597815 10.1016/j.nbd.2023.106264 PMC10494928

[24] Yang, Y. et al. SVCT2 promotes neural stem/progenitor cells migration through activating CDC42 after ischemic stroke. *Front. Cell Neurosci.* **13**, 429 (2019). 31607868 10.3389/fncel.2019.00429 PMC6761321

[25] Jiang, B. et al. Role of glibenclamide in brain injury after intracerebral hemorrhage. *Transl. Stroke Res.* **8**, 183–193 (2017). 27807801 10.1007/s12975-016-0506-2

[26] Li, W. et al. Molecular basis of the acetyltransferase activity of MEC-17 towards α-tubulin. *Cell Res.* **22**, 1707–1711 (2012). 23128673 10.1038/cr.2012.154 PMC3515757

[27] Yang, Y. et al. MEC17-induced α-tubulin acetylation restores mitochondrial transport function and alleviates axonal injury after intracerebral hemorrhage in mice. *J. Neurochem.* **160**, 51–63 (2022). 34407220 10.1111/jnc.15493

[28] Payne, S., De Val, S. & Neal, A. Endothelial-specific Cre mouse models. *Arterioscler. Thromb. Vasc. Biol.* **38**, 2550–2561 (2018). 30354251 10.1161/ATVBAHA.118.309669 PMC6218004

[29] Verheggen, I. C. M. et al. Increase in blood–brain barrier leakage in healthy, older adults. *Geroscience* **42**, 1183–1193 (2020). 32601792 10.1007/s11357-020-00211-2 PMC7394987

[30] Benson, K., Cramer, S. & Galla, H. J. Impedance-based cell monitoring: barrier properties and beyond. *Fluids Barriers CNS* **10**, 5 (2013). 23305242 10.1186/2045-8118-10-5 PMC3560213

[31] Srinivasan, B. et al. TEER measurement techniques for in vitro barrier model systems. *J. Lab Autom.* **20**, 107–126 (2015). 25586998 10.1177/2211068214561025 PMC4652793

[32] Krolak, T. et al. A high-efficiency AAV for endothelial cell transduction throughout the central nervous system. *Nat. Cardiovasc. Res.* **1**, 389–400 (2022). 35571675 10.1038/s44161-022-00046-4 PMC9103166

[33] Butler, K. V. et al. Rational design and simple chemistry yield a superior, neuroprotective HDAC6 inhibitor, tubastatin A. *J. Am. Chem. Soc.* **132**, 10842–10846 (2010). 20614936 10.1021/ja102758v PMC2916045

[34] Petrache, I. et al. The role of the microtubules in tumor necrosis factor-α-induced endothelial cell permeability. *Am. J. Respir. Cell Mol. Biol.* **28**, 574–581 (2003). 12707013 10.1165/rcmb.2002-0075OC

[35] Yu, J. et al. Selective HDAC6 inhibition prevents TNF-α-induced lung endothelial cell barrier disruption and endotoxin-induced pulmonary edema. *Am. J. Physiol. Lung Cell Mol. Physiol.* **311**, L39–L47 (2016). 27190059 10.1152/ajplung.00051.2016

[36] Krendel, M., Zenke, F. T. & Bokoch, G. M. Nucleotide exchange factor GEF-H1 mediates cross-talk between microtubules and the actin cytoskeleton. *Nat. Cell Biol.* **4**, 294–301 (2002). 11912491 10.1038/ncb773

[37] Birukova, A. A. et al. Novel role of microtubules in thrombin-induced endothelial barrier dysfunction. *FASEB J.* **18**, 1879–1890 (2004). 15576491 10.1096/fj.04-2328com

[38] Shang, X. et al. Rational design of small molecule inhibitors targeting RhoA subfamily Rho GTPases. *Chem. Biol.* **19**, 699–710 (2012). 22726684 10.1016/j.chembiol.2012.05.009 PMC3383629

[39] Birukova, A. A. et al. GEF-H1 is involved in agonist-induced human pulmonary endothelial barrier dysfunction. *Am. J. Physiol. Lung Cell Mol. Physiol.* **290**, L540–L548 (2006). 16257999 10.1152/ajplung.00259.2005

[40] Zhao, M. J. et al. Roles of RAGE/ROCK1 pathway in HMGB1-induced early changes in barrier permeability of human pulmonary microvascular endothelial cell. *Front. Immunol.* **12**, 697071 (2021). 34745088 10.3389/fimmu.2021.697071 PMC8564108

[41] Birukova, A. A. et al. Involvement of microtubules and Rho pathway in TGF-β1-induced lung vascular barrier dysfunction. *J. Cell. Physiol.* **204**, 934–947 (2005). 15828024 10.1002/jcp.20359

[42] Birukova, A. A. et al. Microtubule disassembly induces cytoskeletal remodeling and lung vascular barrier dysfunction: role of Rho-dependent mechanisms. *J. Cell Physiol.* **201**, 55–70 (2004). 15281089 10.1002/jcp.20055

[43] Enomoto, T. Microtubule disruption induces the formation of actin stress fibers and focal adhesions in cultured cells: possible involvement of the rho signal cascade. *Cell Struct. Funct.* **21**, 317–326 (1996). 9118237 10.1247/csf.21.317

[44] Verin, A. D. et al. Microtubule disassembly increases endothelial cell barrier dysfunction: role of MLC phosphorylation. *Am. J. Physiol. Lung Cell Mol. Physiol.* **281**, L565–L574 (2001). 11504682 10.1152/ajplung.2001.281.3.L565

[45] Smurova, K. M. et al. The microtubule system in endothelial barrier dysfunction: disassembly of peripheral microtubules and microtubules reorganization in internal cytoplasm. *Tsitologiia* **50**, 49–55 (2008). 18409368

[46] Carmona, B. et al. Tubulin post-translational modifications: the elusive roles of acetylation. *Biology* **12**, 561 (2023). 10.3390/biology12040561 PMC10136095 37106761

[47] Feng, W. et al. SUMOylation of α-tubulin is a novel modification regulating microtubule dynamics. *J. Mol. Cell Biol.* **13**, 91–103 (2021). 33394042 10.1093/jmcb/mjaa076 PMC8104938

[48] Nieuwenhuis, J. & Brummelkamp, T. R. The tubulin detyrosination cycle: function and enzymes. *Trends Cell Biol.* **29**, 80–92 (2019). 30213517 10.1016/j.tcb.2018.08.003

[49] Li, L. et al. MEC-17 deficiency leads to reduced α-tubulin acetylation and impaired migration of cortical neurons. *J. Neurosci.* **32**, 12673–12683 (2012). 22972992 10.1523/JNEUROSCI.0016-12.2012 PMC6703811

[50] Neumann, B. & Hilliard, M. A. Loss of MEC-17 leads to microtubule instability and axonal degeneration. *Cell Rep.* **6**, 93–103 (2014). 24373971 10.1016/j.celrep.2013.12.004 PMC3939029

[51] Bruhn, P. J. et al. Tubastatin A prevents hemorrhage-induced endothelial barrier dysfunction. *J. Trauma Acute Care Surg.* **84**, 386–392 (2018). 29194316 10.1097/TA.0000000000001753 PMC5780204

[52] Yuan, Y. G., Peng, Q. L. & Gurunathan, S. Combination of palladium nanoparticles and tubastatin-A potentiates apoptosis in human breast cancer cells: a novel therapeutic approach for cancer. *Int. J. Nanomed.* **12**, 6503–6520 (2017). 10.2147/IJN.S136142 PMC5592949 28919751

[53] Ren, Y. et al. Cloning and characterization of GEF-H1, a microtubule-associated guanine nucleotide exchange factor for Rac and Rho GTPases. *J. Biol. Chem.* **273**, 34954–34960 (1998). 9857026 10.1074/jbc.273.52.34954

[54] Tian, X. et al. Novel role of stathmin in microtubule-dependent control of endothelial permeability. *FASEB J.* **26**, 3862–3874 (2012). 22700873 10.1096/fj.12-207746 PMC3425818

[55] Jalimarada, S. S. et al. Microtubule disassembly breaks down the barrier integrity of corneal endothelium. *Exp. Eye Res.* **89**, 333–343 (2009). 19345211 10.1016/j.exer.2009.03.019 PMC2745835

[56] Chu, X. et al. Coupling between interleukin-1R1 and necrosome complex involves in hemin-induced neuronal necroptosis after intracranial hemorrhage. *Stroke* **49**, 2473–2482 (2018). 30355103 10.1161/STROKEAHA.117.019253

[57] Duan, L. et al. Baicalin inhibits ferroptosis in intracerebral hemorrhage. *Front. Pharm.* **12**, 629379 (2021). 10.3389/fphar.2021.629379 PMC8017143 33815110

[58] Graca-Souza, A. V. et al. Neutrophil activation by heme: implications for inflammatory processes. *Blood* **99**, 4160–4165 (2002). 12010821 10.1182/blood.v99.11.4160

[59] Nakaya, Y. et al. RhoA and microtubule dynamics control cell–basement membrane interaction in EMT during gastrulation. *Nat. Cell Biol.* **10**, 765–775 (2008). 18552836 10.1038/ncb1739

