# Supplementary material for: Acetylation of α-tubulin restores endothelial cell injury and blood–brain barrier disruption after intracerebral hemorrhage in mice
Source: Exp Mol Med. 2025 May 7;57(5):1064–77. doi: 10.1038/s12276-025-01454-9 (PMC12130200; doi:10.1038/s12276-025-01454-9)
Supplement: Supplementary file 1 — Supplementary Information 1 [file 12276_2025_1454_MOESM1_ESM.pdf]

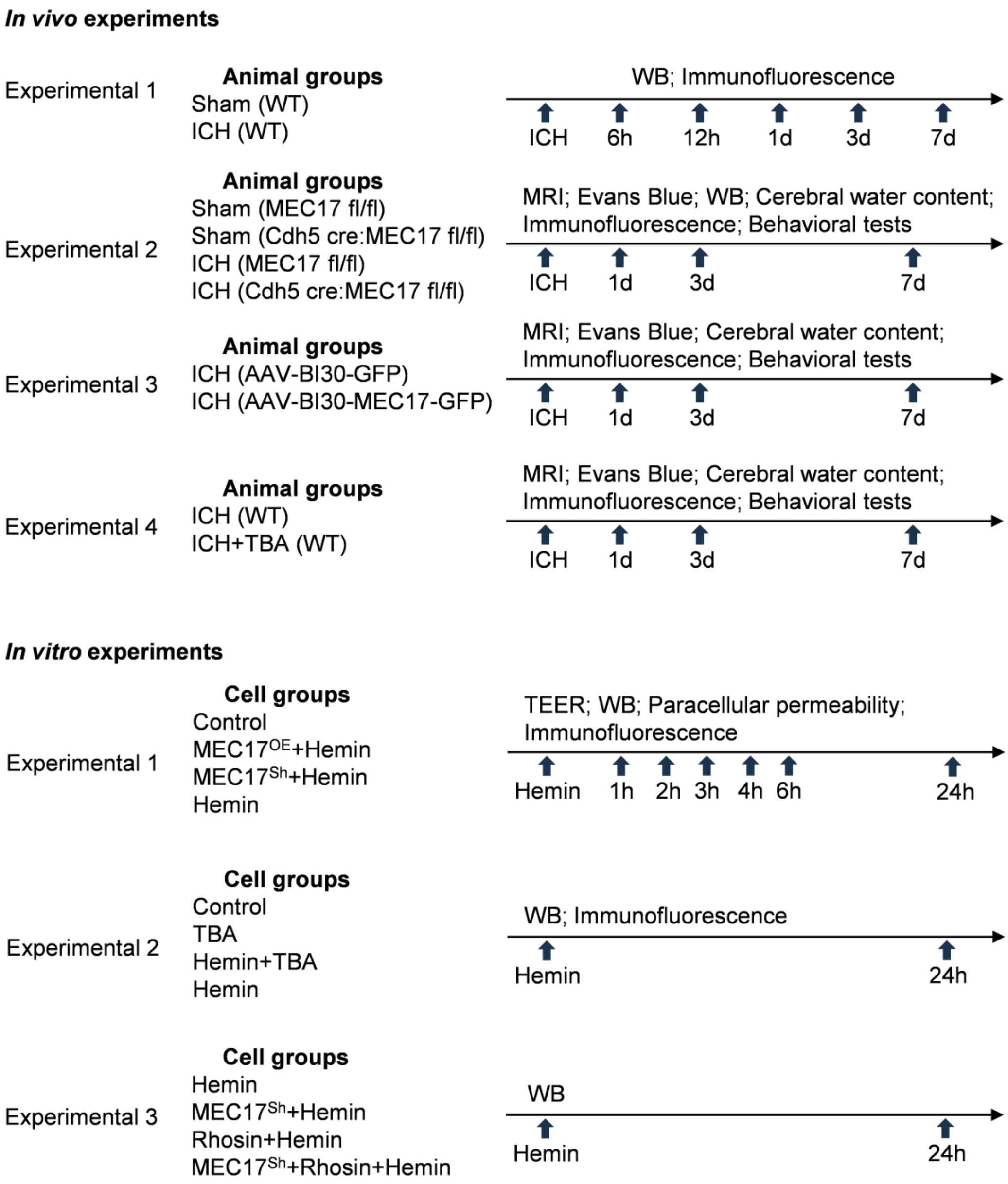

Supplementary Fig. 1. Experiments design.

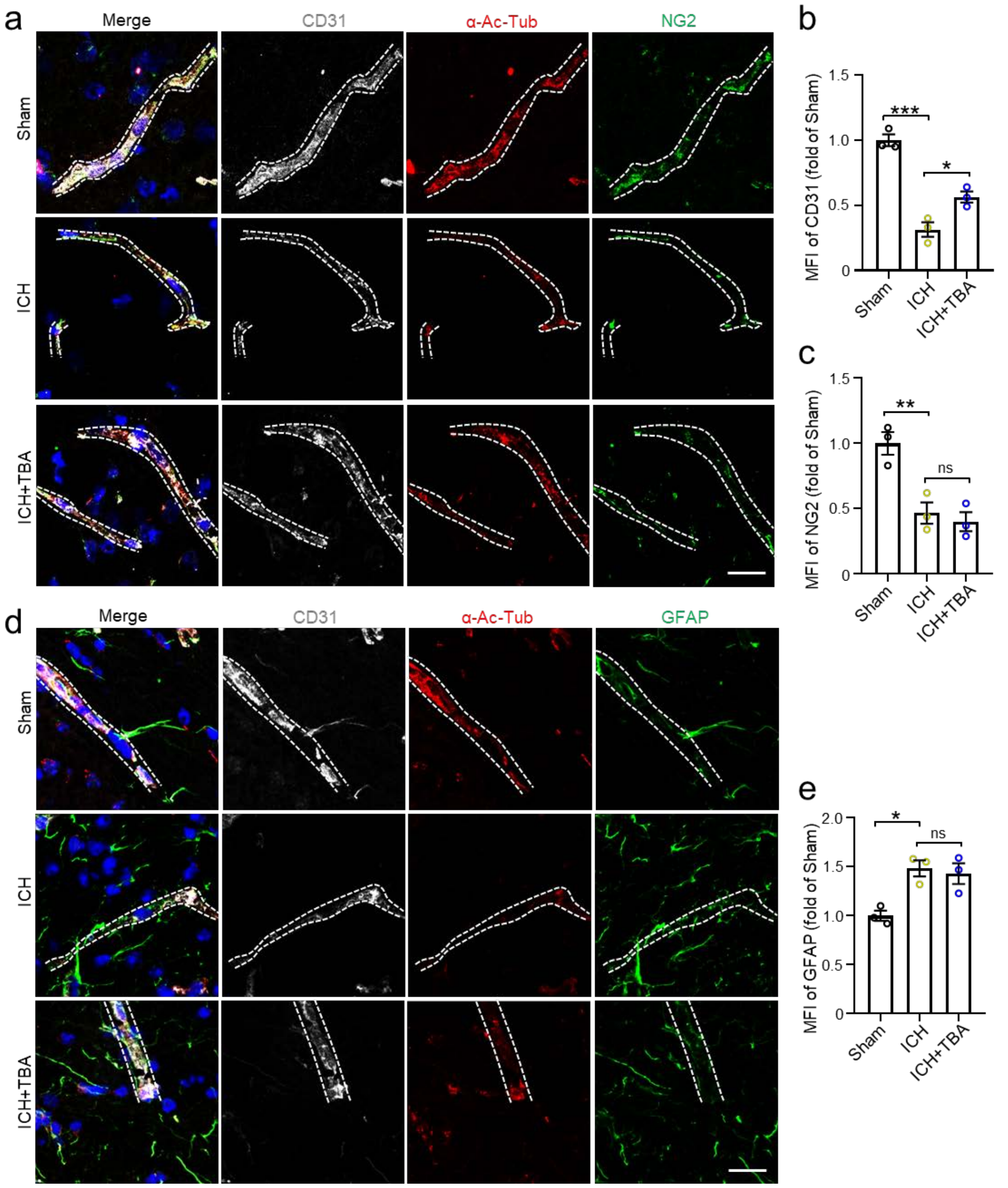

Supplementary Fig. 2. α-Ac-Tub did not affect pericytes and astrocytes in the neurovascular unit after ICH. a, Representative immunofluorescence pictures of CD31 (white), α-Ac-Tub (red) and NG2 (green) around hematoma in each group at day 3 after ICH. Scale bar, 20 µm. **b,** Statistical analysis of CD31 along the vessels around hematoma in each group. MFI of CD31 was quantified and expressed relative to the Sham group (n = 3 animals per group). **c,** Statistical analysis of NG2 along the vessels around hematoma in each group. MFI of NG2 was quantified and expressed relative to the Sham group (n = 3 animals per group). **d,** Representative immunofluorescence pictures of CD31 (white), α-Ac-Tub (red) and GFAP (green) around hematoma in each group at day 3 after ICH. Scale bar, 20 µm. **e,** Statistical analysis of GFAP along the vessels around hematoma in each group. MFI of GFAP was quantified and expressed relative to the Sham group (n = 3 animals per group). Data are

shown as the mean ± SEM. ***$P<0.001$, **$P<0.01$, *$P<0.05$, ns=not significant. One-way ANOVA followed by the Tukey’s post hoc test for b, c, and e.

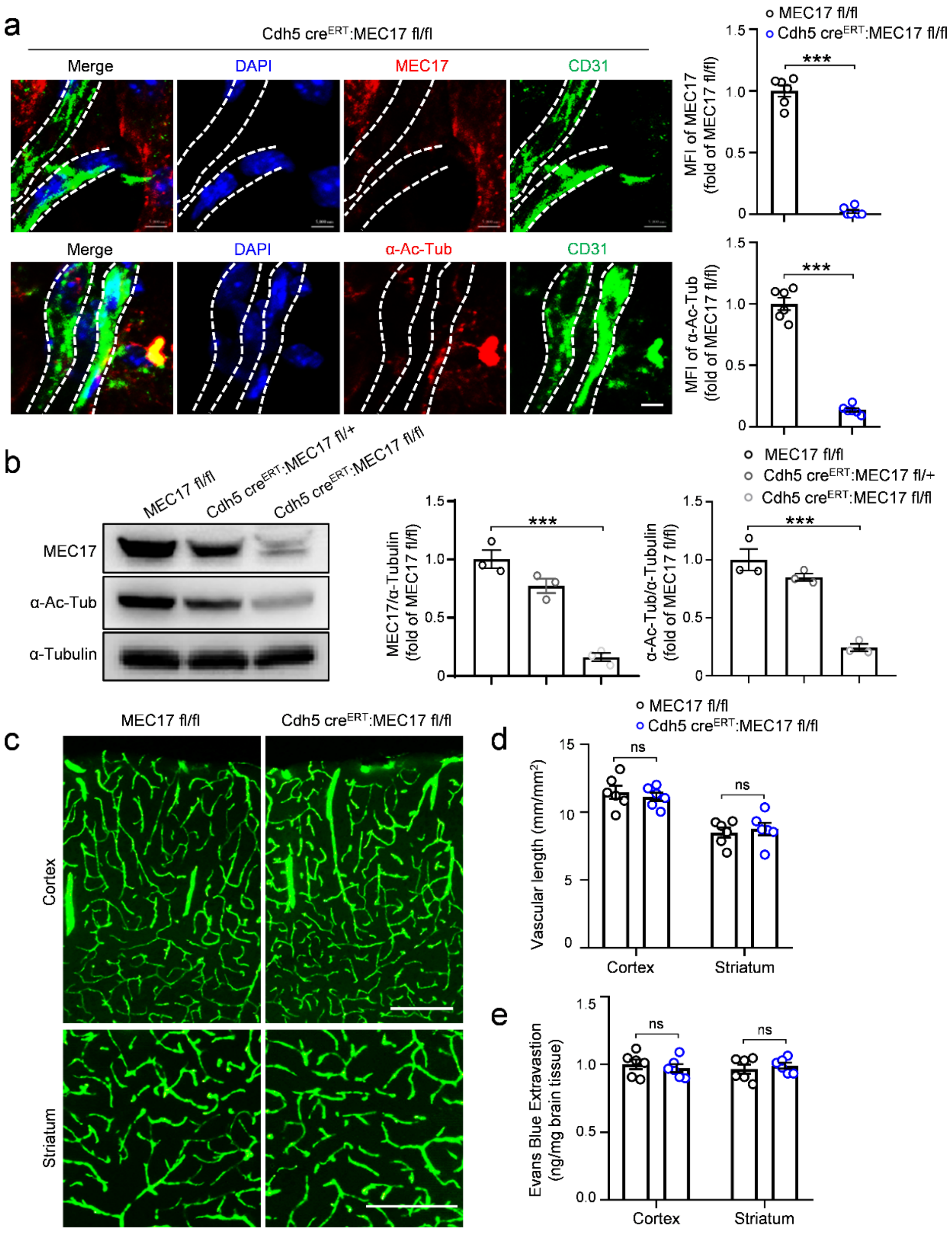

Supplementary Fig. 3. Validation of conditional knockout MEC17 in ECs. a, Representative immunofluorescence pictures and statistical analysis of MEC17 (red, up), α-Ac-Tub (red, down) and CD31 (green) in the brains of each group. MFI of MEC17 and α-Ac-Tub was quantified and expressed relative to the MEC17$^{fl/fl}$ group (n = 6 animals per group). Scale bar, 5 μm. **b,** Expression of MEC17 and α-Ac-Tub were evaluated in brains by western blotting in each group. α-Tubulin was used as an internal loading control. Blots of MEC17 and α-Ac-Tub were quantified and expressed relative to the MEC17$^{fl/fl}$ group (n = 3 animals per group). **c,** Representative immunofluorescence pictures of dextran-GFP perfused

blood vessels in cortex and striatum of each group. Scale bar, 100 μm. **d,** The length of blood vessels in cortex and striatum were quantified per mm^2 in each group (n = 6 animals per group). **e,** Evans blue extravasation in cortex and striatum was measured using spectrophotometer in each group (n = 6 animals per group). Data are shown as the mean ± SEM. ***P<0.001, ns=not significant. Two-tailed Student's t-tests for a, d and e. One-way ANOVA followed by the Tukey’s post hoc test for b.

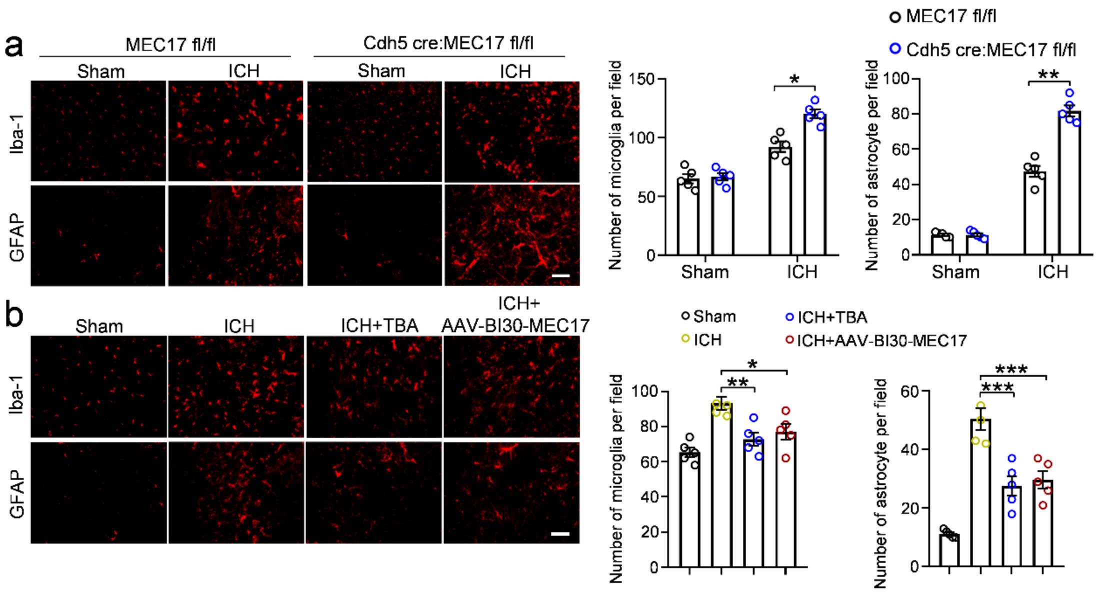

Supplementary Fig. 4. α-Ac-Tub overexpression inhibits the activation of microglia and astrocytes after ICH. a, Representative immunofluorescence pictures and statistical analysis of Iba-1 (up) and GFAP (down) around hematoma in each group after ICH. Number of Iba-1 positive microglia and GFAP positive astrocytes were quantified per field (n = 5 animals per group). Scale bar, 10 μm. **b,** Representative immunofluorescence pictures and statistical analysis of Iba-1 (up) and GFAP (down) around hematoma in each group. Number of Iba-1 positive microglia and GFAP positive astrocytes were quantified per field (n = 5 animals per group). Scale bar, 10 μm. Data are shown as the mean ± SEM. **P*<0.05, ***P*<0.01, ****P*<0.001, ns=not significant. Two-way ANOVA followed by the Tukey's post hoc test for a. One-way ANOVA followed by the Tukey's post hoc test for b.

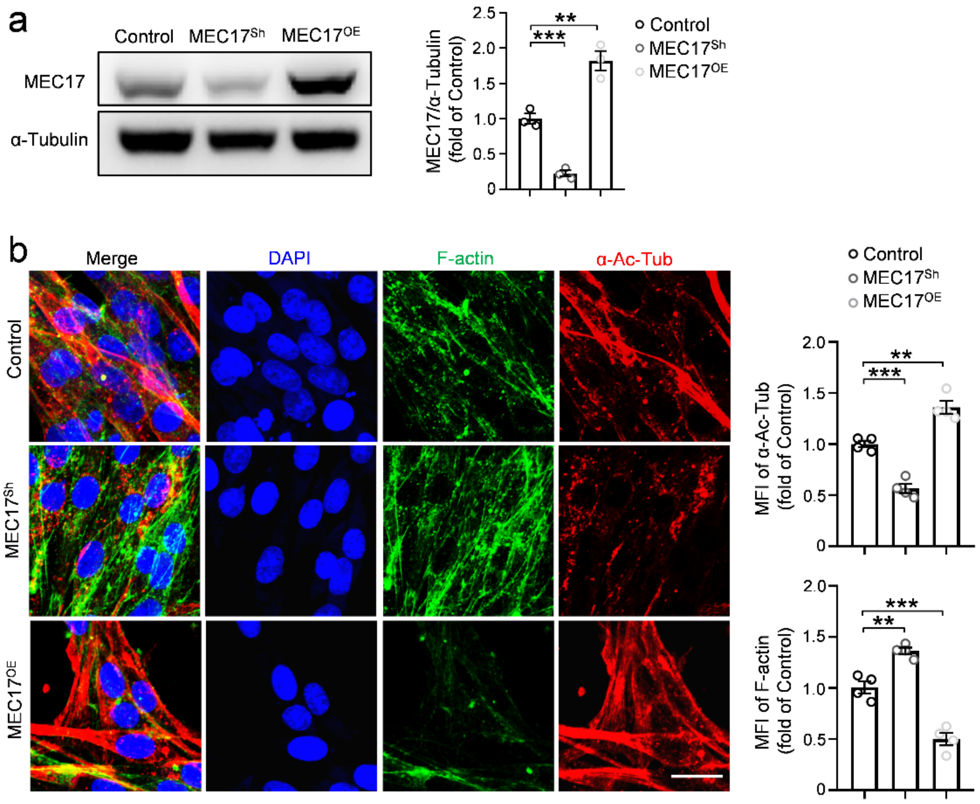

Supplementary Fig. 5. Expression of α-Ac-Tub and F-actin after MEC17 overexpression and silencing. a, Expression of MEC17 was evaluated in the brains by western blotting after overexpression and silencing. α-Tubulin was used as an internal loading control. Blots of MEC17 were quantified and expressed relative to the Control group (n = 3 animals per group). **b,** Representative immunofluorescence pictures and statistical analysis of F-actin (green) and α-Ac-Tub (red) in HBMECs after MEC17 overexpression or silencing. MFI of α-Ac-Tub and F-actin was quantified and expressed relative to the Control group (n = 4 independent culture per group). Scale bar, 20 μm. Data are shown as the mean ± SEM. $^{**}P<0.01$ and $^{***}P<0.001$. One-way ANOVA followed by the Tukey's post hoc test.

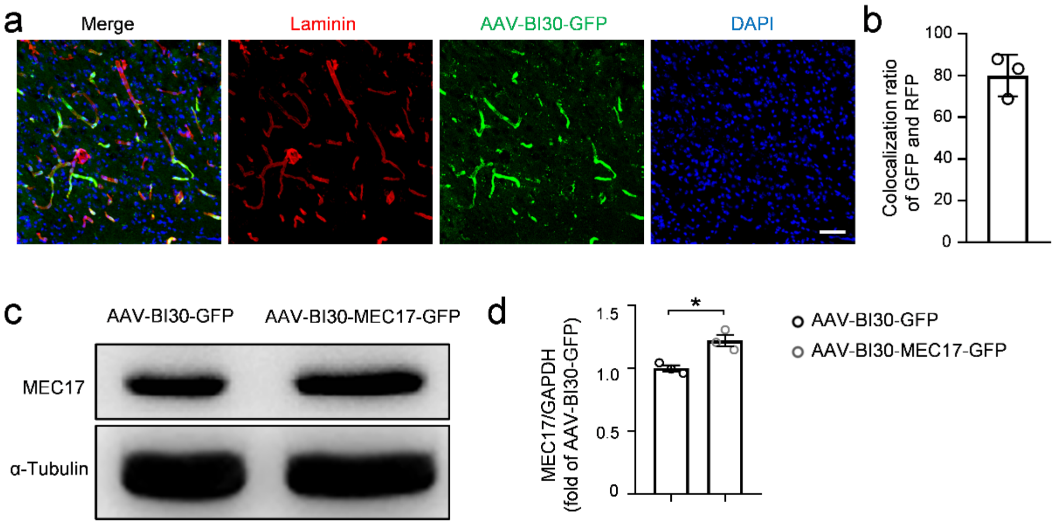

Supplementary Fig. 6. Validation of conditional overexpression MEC17 in ECs by using AAV-BI30. a, Representative immunofluorescence pictures of Laminin (red) and AAV-BI30-GFP (green). Scale bar, 50 µm. **b,** Colocalization of GFP and RFP was quantified (n = 3 animals per group). **c,** Expression of MEC17 was evaluated in the brains by western blotting at 3 weeks after AAV-BI30-MEC17^{OE}-GFP injection. **d,** Blots of MEC17 were quantified and expressed relative to the AAV-BI30-GFP group (n = 3 animals per group). Data are shown as the mean ± SEM. *P<0.05. Two-tailed Student's t-tests.
