# Supplementary material for: Acetylation of α-tubulin restores endothelial cell injury and blood–brain barrier disruption after intracerebral hemorrhage in mice
Source: Exp Mol Med. 2025 May 7;57(5):1064–77. doi: 10.1038/s12276-025-01454-9 (PMC12130200; doi:10.1038/s12276-025-01454-9)

Full, unedited Western blot images of Fig. 1a

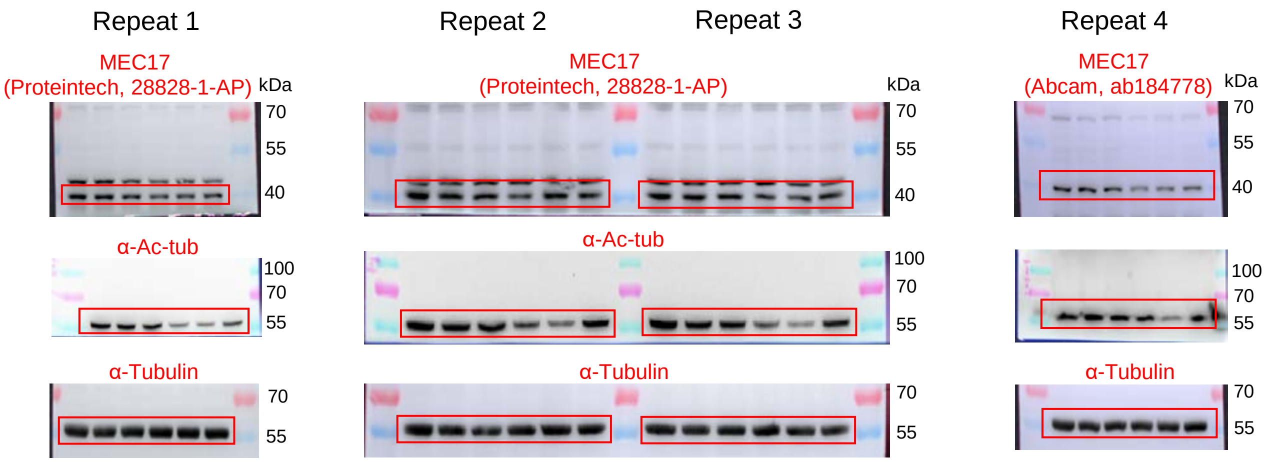

Full, unedited Western blot images of Fig. 2e

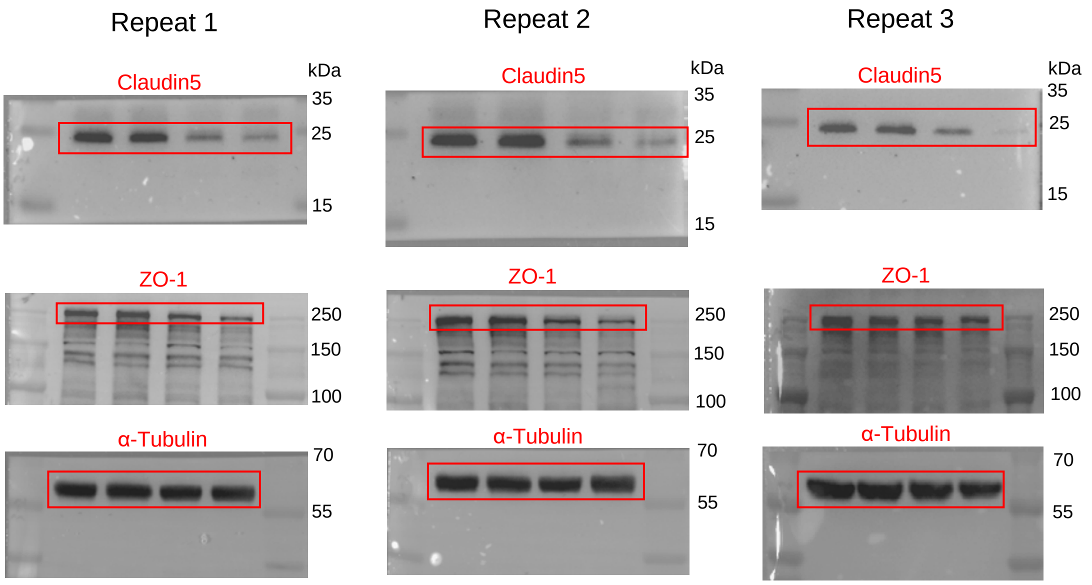

Full, unedited Western blot images of Fig. 3e

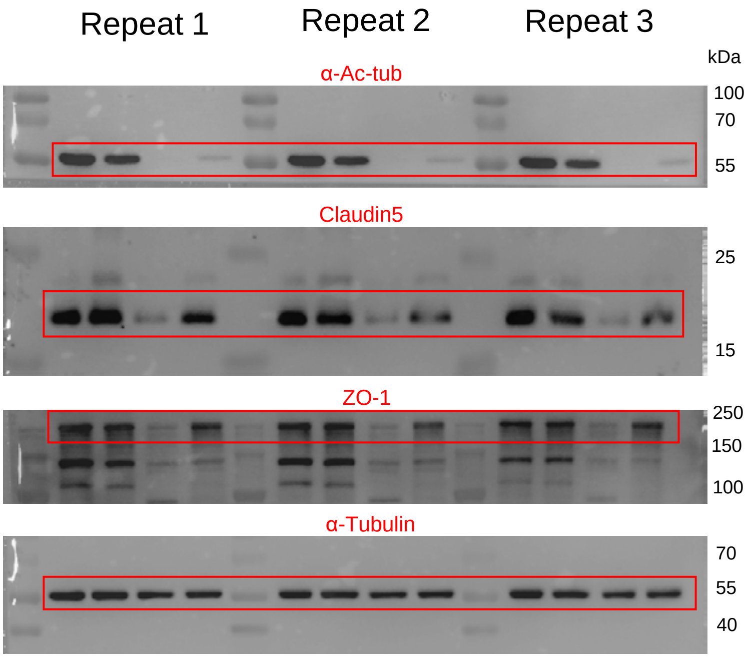

Full, unedited Western blot images of Fig. 5b

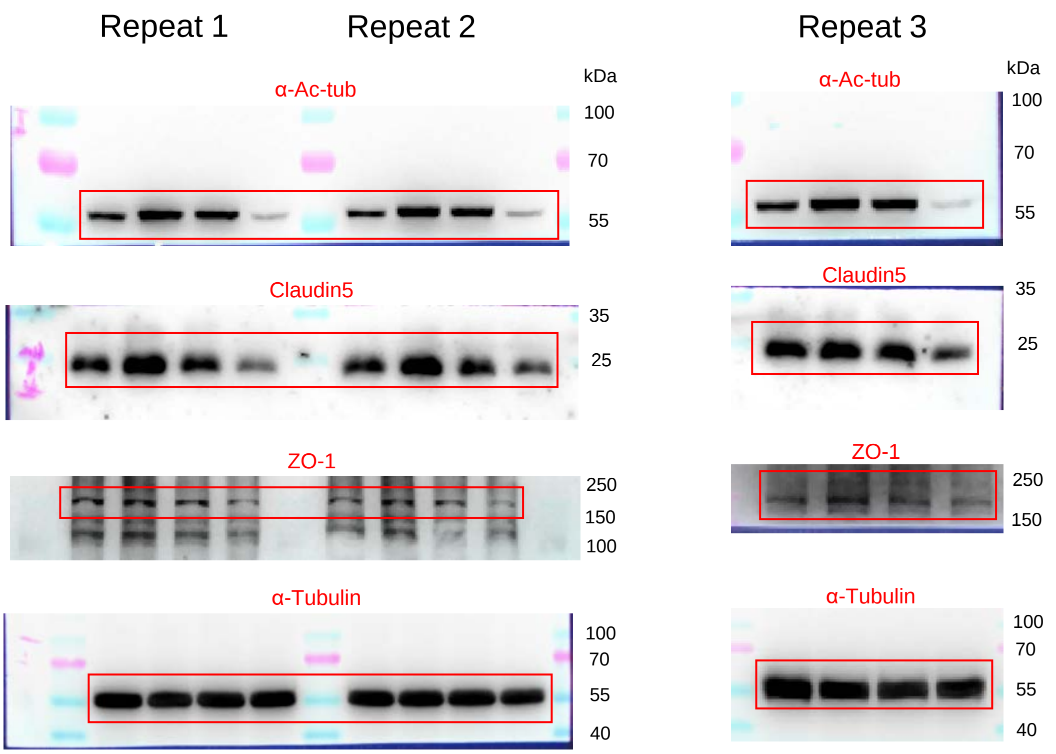

Full, unedited Western blot images of Fig. 6a

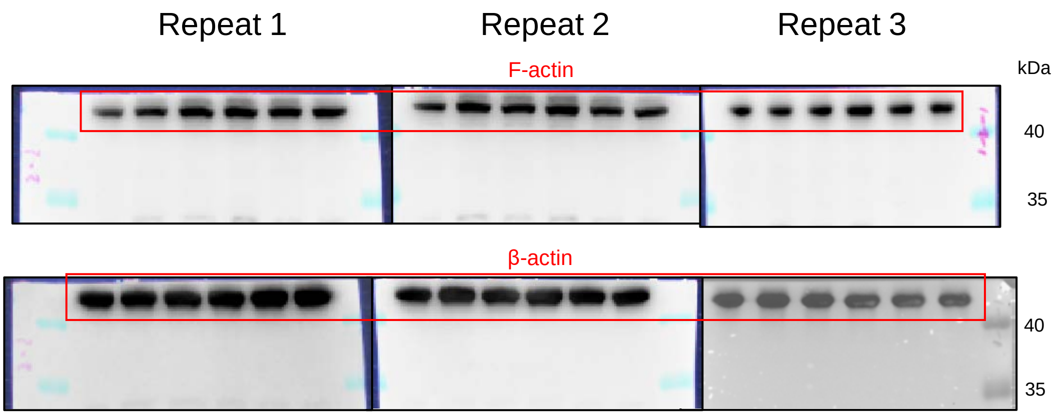

Full, unedited Western blot images of Fig. 6g

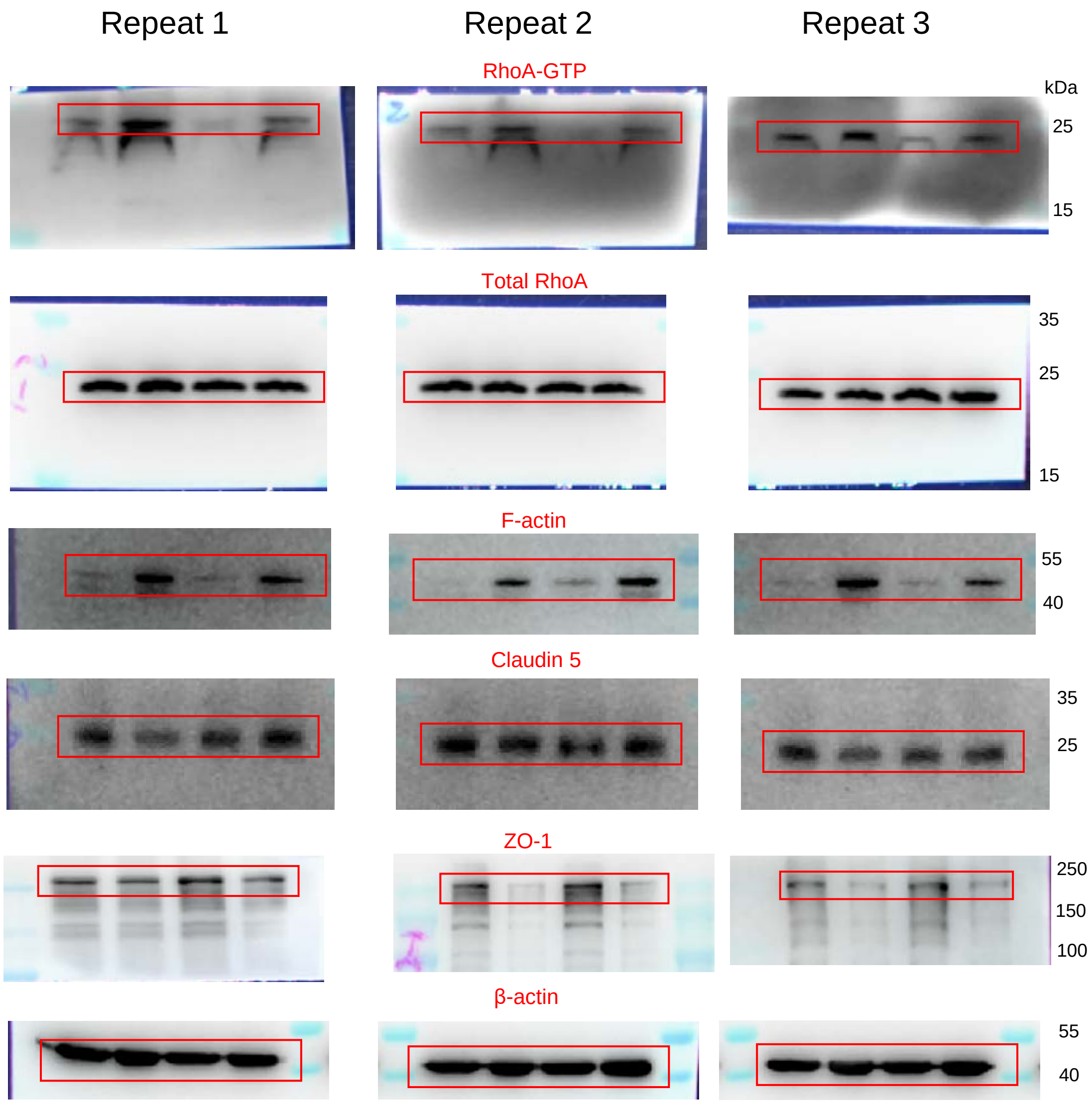

Full, unedited Western blot images of Supplementary Fig. 3b

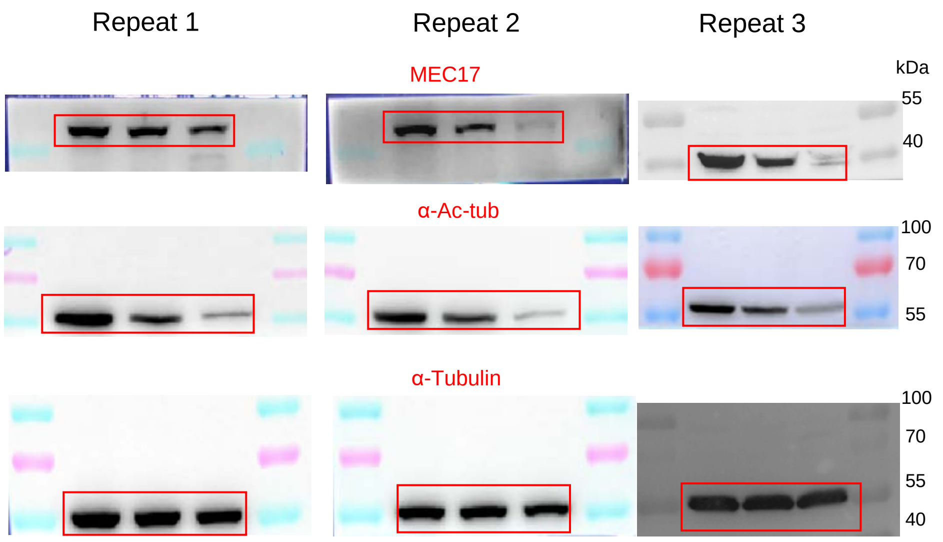

Full, unedited Western blot images of Supplementary Fig. 5a

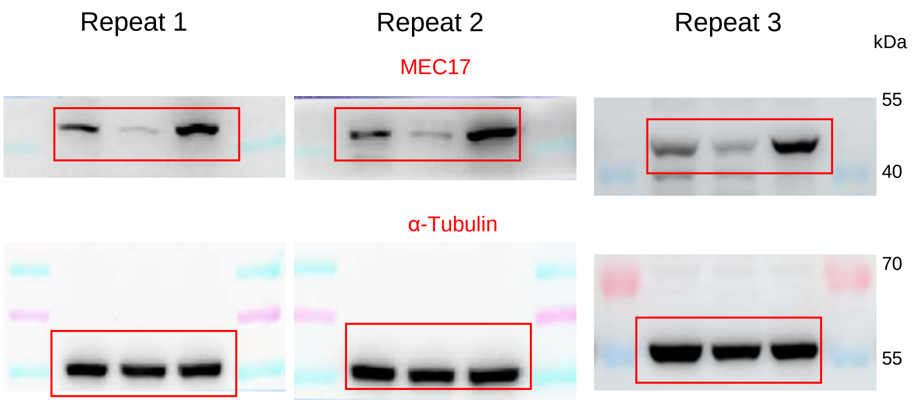

Full, unedited Western blot images of Supplementary Fig. 6c

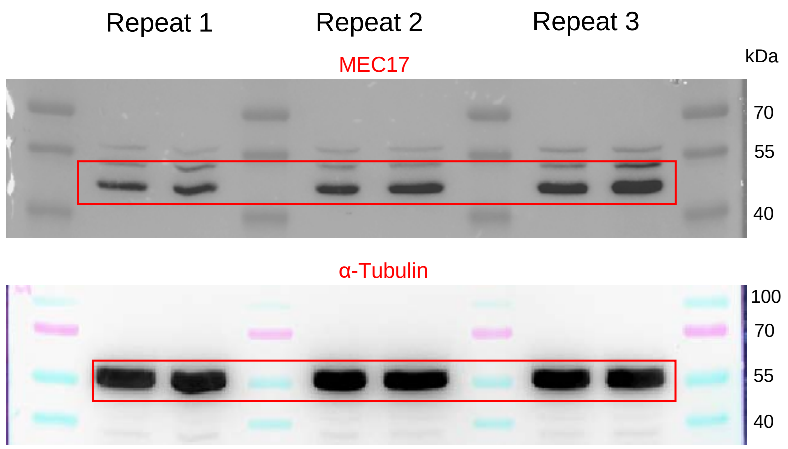

Supplement: Supplementary file 2 — Supplementary Information 2 [file 12276_2025_1454_MOESM2_ESM.pdf]
